# The draft genome of a wild barley genotype reveals its enrichment in genes related to biotic and abiotic stresses compared to cultivated barley

**DOI:** 10.1111/pbi.13210

**Published:** 2019-08-04

**Authors:** Miao Liu, Yan Li, Yanling Ma, Qiang Zhao, Jiri Stiller, Qi Feng, Qilin Tian, Dengcai Liu, Bin Han, Chunji Liu

**Affiliations:** ^1^ CSIRO Agriculture and Food St Lucia Qld Australia; ^2^ Crop Research Institute Sichuan Academy of Agricultural Sciences Jinjiang District, Chengdu China; ^3^ Triticeae Research Institute Sichuan Agricultural University Wenjiang, Chengdu China; ^4^ National Center for Gene Research Chinese Academy of Sciences Shanghai China; ^5^ Institute of Crop Sciences Chinese Academy of Agricultural Sciences Haidian District, Beijing China

**Keywords:** *Hordeum spontaneum*, genome, Morex, specific gene, genetic variation

## Abstract

Wild barley (*Hordeum spontaneum*) is the progenitor of cultivated barley (*Hordeum vulgare*) and provides a rich source of genetic variations for barley improvement. Currently, the genome sequences of wild barley and its differences with cultivated barley remain unclear. In this study, we report a high‐quality draft assembly of wild barley accession (AWCS276; henceforth named as WB1), which consists of 4.28 Gb genome and 36 395 high‐confidence protein‐coding genes. BUSCO analysis revealed that the assembly included full lengths of 95.3% of the 956 single‐copy plant genes, illustrating that the gene‐containing regions have been well assembled. By comparing with the genome of the cultivated genotype Morex, it is inferred that the WB1 genome contains more genes involved in resistance and tolerance to biotic and abiotic stresses. The presence of the numerous WB1‐specific genes indicates that, in addition to enhance allele diversity for genes already existing in the cultigen, exploiting the wild barley taxon in breeding should also allow the incorporation of novel genes. Furthermore, high levels of genetic variation in the pericentromeric regions were detected in chromosomes 3H and 5H between the wild and cultivated genotypes, which may be the results of domestication. This *H. spontaneum* draft genome assembly will help to accelerate wild barley research and be an invaluable resource for barley improvement and comparative genomics research.

## Introduction

To breed varieties to meet the needs of changing biotic and abiotic stresses or of new foods with nutritional and health benefits demanded by consumers, plant breeders require consistent access to new genetic variations. Growth in global food demand and the potential impact of climate change have increased the pressure for effective crop breeding. As available genetic variations among elite varieties for most crop species are often limited, exploiting close relatives of domesticated plant species has provided a source of genes that have allowed significant advances in crop productivity (Brozynska *et al.*, 2016; Hajjar and Hodgkin, 2007). It was estimated that about 30% of increased crop yields in the late 20th century, valued worldwide at around US $100 billion, can be attributed to the use of close relatives in plant breeding programmes (Pimentel *et al.*, 1997).

Barley (*Hordeum vulgare*) is one of the major cereals worldwide and is among the oldest domesticated crops. It was selected from its wild progenitor *H*. *spontaneum.* Wild and domesticated barley differ in several phenotypic characteristics, collectively referred to as the domestication syndrome (Paterson *et al.*, 1995). Both taxa are diploid (2*n* = 2*x* = 14) and predominantly self‐pollinated, and there are no barriers in generating fully fertile progeny between them (Zohary and Hopf, 2000). Previous studies showed that only some 40% of the alleles found in wild barley are present in cultivars and wild barley is thus a rich source of genetic variations for various breeding programmes (Ellis *et al.*, 2000; Horns and Hood, 2012; Tombuloglu *et al.*, 2015). A wide range of traits in wild barley have been assessed, and they include resistance and tolerance to different biotic (Abbott *et al.*, 1991; Biselli *et al.*, 2010; Chen *et al.*, 2013; Friedt *et al.*, 2011; Schmalenbach *et al.*, 2008) and abiotic (Kalladan *et al.*, 2013; Lakew *et al.*, 2013; Newton *et al.*, 2011; Pakniyat and Namayandeh, 2007; Russell *et al.,* 2014; Shavrukov *et al.*, 2010; Wang *et al.*, 2018) stresses, as well as variations for grain quality (Batchu and Zimmermann, 2006; Ellis *et al.*, 1993; Erkkilä *et al.*, 1998; Jun *et al.*, 2011; Li *et al.*, 2010). The existence of genetic variation is the foundation for crop improvement. The rate of breeding success, however, also depends on the availability of methods for efficient detection, transfer and integration of targeted genes in breeding programmes (Brozynska *et al.*, 2016). The availability of high‐quality genome sequences of barley would be a prerequisite for developing such methods (Dai *et al.*, 2018; Mascher *et al.*, 2017; Sato *et al.*, 2015; The International Barley Genome Sequencing Consortium, 2012; Zeng *et al.*, 2015). In the study reported here, we report a high‐quality draft genome sequence of a wild barley accession (AWCS276; henceforth named as WB1), which was collected from Iran, one of the main distribution centres and origin regions of wild barley (Salamini *et al.*, 2002; Zohary *et al.*, 2012). Differences between this WB1 genome and that of the international reference genome based on the cultivated genotype Morex were described in this publication.

## Results

### Assembly of the WB1 genome sequences and repeat annotation

The genome size of WB1 was estimated to be about 4.60 ± 0.07 Gb based on the flow cytometric analysis (Figure S2A). Results from the kmerFreq‐AR showed that the genome sizes of the WB1 and Morex were about 4.45 and 4.76 Gb, respectively (Figure S2B). Based on the International Barley Sequencing Consortium (2012), the genome size of Morex is 5.1 Gb. These results all show that the WB1 genome was slightly smaller than that of Morex.

In total, 705.41 Gb of Illumina raw reads were obtained from WB1 (Table S1). These sequences were assembled using a combined strategy (Figure S1), which allowed the generation of a high‐quality draft genome with a total length of 4.28 Gb with 0.27 Gb gaps in Ns. N50 lengths of the assembled sequence contigs and scaffolds were 35.4 and 724.9 kb, respectively (Table 1 and Table S3). Of the 956 single‐copy Benchmarking Universal Single‐Copy Orthologs (BUSCOs), 95.3% were found to be complete, while only 2.6% were missing in the WB1 assembly. The corresponding percentages for Morex were 93.0% and 4.0%, respectively. The quality of our assembly was of good quality in comparison with other plant species (Table S4). When analysed against the 28 620 fl‐cDNAs of barley available in the public domain, 90.4% of them were significantly matched to the WB1 genome assembly. These results all indicate that the WB1 genome assembly is of high quality and it covers most gene space.

**Table 1:** Statistics of WB1 genome assembly and gene prediction

	Number	Size
Assembly feature		
Estimated genome size		4.60 Gb
Total size of assembled scaffolds		4.28 Gb
N50 (scaffolds)		724 931 bp
N80 (scaffolds)		299 856 bp
Number of scaffolds (>N80 length)	4412	
Longest scaffold		4 913 581 bp
Genome annotation		
Total repetitive sequence		3 330 988 248 bp
Gene models	36 395	45 664 619 bp
Non‐coding RNAs	2777	277 685 bp

Repetitive sequences accounted for 77.8% of the assembled WB1 genome. This number is slightly smaller than that of the Morex (80.8%) (Mascher *et al.*, 2017). The repetitive sequences in WB1 mainly consist of various types of transposable elements (TEs, 99.7%) with LTRs dominating (Table S5). The distribution of the divergence rates (percentage of substitutions in the matching region compared with consensus repeats in constructed libraries) for the TEs peaked at 9% in WB1 and at 8% in Morex, suggesting a more recent expansion of these elements in the latter (Figure S3). Analysing the insertion time of LTRs found that WB1 and Morex started to diverge at about 1–2 million years ago (MYA) and that LTRs mostly inserted to the cultivated barley genome during the last 10 000 years (Figure S4).

### Gene annotation and tissue specificity in the WB1 genome

EvidenceModeler (EVM) annotation pipeline generated a consensus of 88 955 genes from the WB1 sequences based on *de novo* prediction, homology annotation and RNA‐seq prediction. According to the filtering criteria supported by gene evidence, a total of 36 395 high‐confidence protein‐coding genes were retained. Of these gene models, 29 681 (81.6%) were supported by homologous and RNA‐seq expression evidence, 5891 (16.2%) were supported by homologous evidence only, and 823 (2.2%) were supported by RNA‐seq expression evidence only (Figure S5A). The coverage distribution of the WB1 genes supported by homologous or RNA‐seq expression evidence showed that the coverage rates of more than 78% of genes were above 90% (Figure S5B). Functional annotations for 31 813 (87%) of these genes were obtained by InterProScan (Figure S5C).

Transcriptomic analysis detected tissue‐specific and highly expressed genes for each of the six tissues assessed. The numbers of such genes varied from 304 (for seedling) to 1977 (for root) (Figure S6). The leaf‐specific and highly expressed genes were enriched for ‘photosynthesis’, ‘phosphorus metabolic process’ and ‘macromolecule modification’. The root‐specific ones were enriched for stress responses such as ‘response to oxidative stresses’ and ‘response to stimulus’. The spikelet‐specific ones were enriched for ‘carbohydrate metabolic process’, ‘lipid metabolic process’ and ‘transmembrane transport’. The developing kernel‐specific ones were enriched with ‘cellular modified amino acid metabolic process’ and ‘oxidation‐reduction process’. The stem‐specific ones were enriched for ‘lipid transport’ and ‘translation’ (Figure S7).

A total of 1920 tRNA (Table S6), 163 small nucleolar RNA (CD‐box, HACA‐box), 136 small nuclear RNA (snRNA) and 558 microRNA (miRNA) genes (Table S7) were identified. Based on the PlantTFDB pipeline, 2049 transcription factors (TFs) were identified and they were classified into 56 families. The number of each TF family in the WB1 genome was similar to other related grass genomes (Figure S8).

### Phylogenetic relationship, divergence time and gene expansion/contraction between wild barley and other species

**Figure 1:**
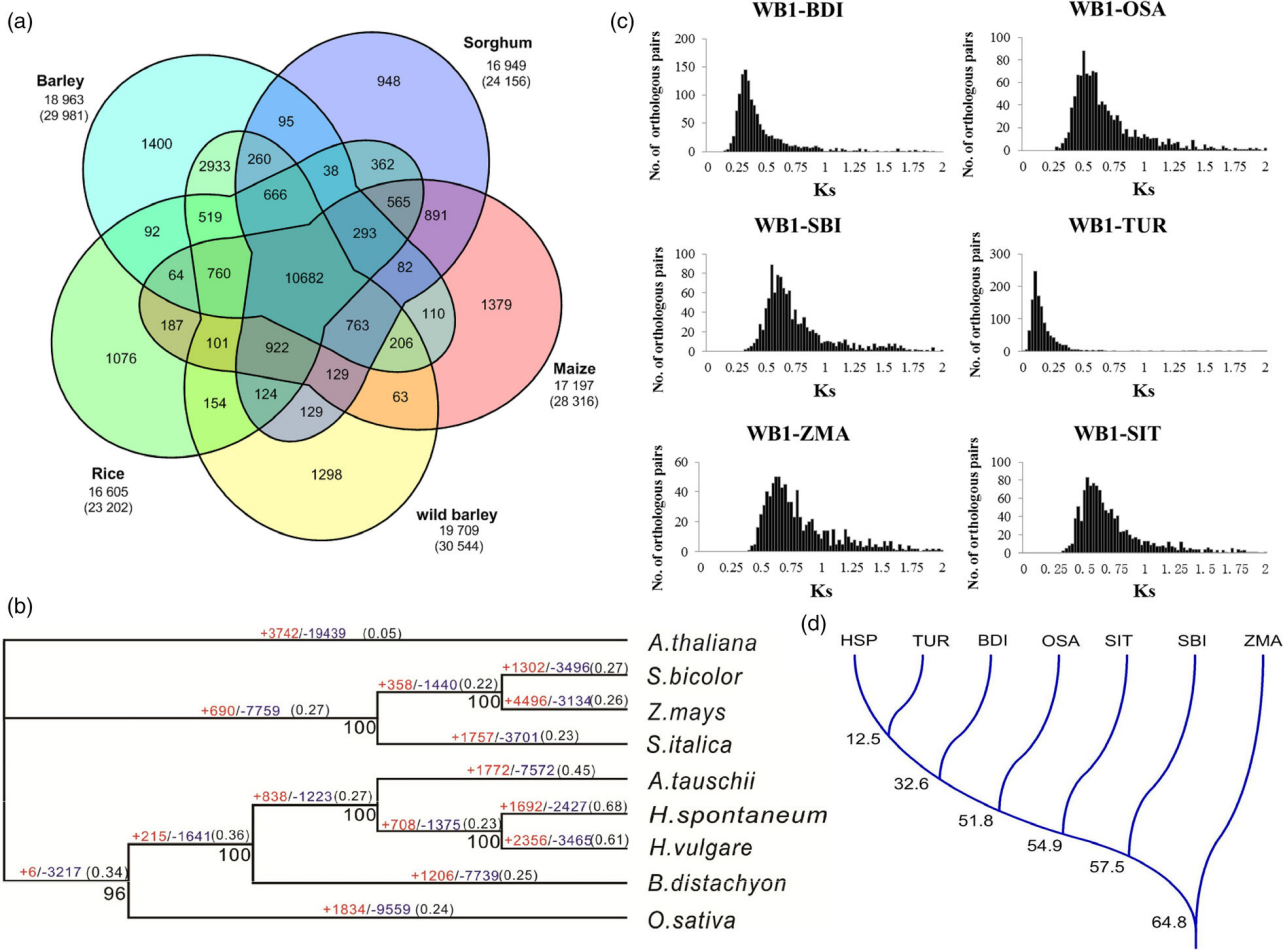
Gene families, phylogenetic, *K*s distributions and divergence time between wild barley and other grasses. (a) Venn diagram of shared orthologous gene families among five grass genomes. The first number below the species name denotes the number of gene families clustered by OrthoMCL analysis. The second number indicates the number of genes within families for each taxon. (b) Phylogenetic relationship between the wild barley taxon and other grasses. The numbers in red and blue on each branch indicate the quantity of expanded (+) or contracted (−) orthologous clusters after the corresponding speciation, respectively. The tree is based on 100 bootstraps shown by black numbers. The dN/dS ratio of each branch is noted in parentheses. *Arabidopsis* *thaliana* is used as an outgroup. (c) *K*s distributions of orthologous genes between wild barley genotype WB1 and other four grass species. (d) Divergence time between wild barley and other grasses. HSP: *H. spontaneum*; TUR: *T. urartu*; BDI: *B. distachyon*; OSA: *O. sativa*; SIT: *S. italica*; SBI: *S. bicolor*; ZMA: *Z. mays*.

To establish phylogenetic relationship between wild barley and other species (including *Arabidopsis thaliana*, *Sorgum bicolor*, *Zea mays*, *Setariai talica*, *Aegilops tauschii*, *Brachypodium distachyon*, *Triticum urartu, Oryza sativa* and *H. vulgare*), 296 911 coding sequences from these species were assessed. These coding sequences were clustered into 29 977 gene families. Comparing the gene families among the five grass genomes (including *S. bicolor*, *Z. mays*, *O. sativa*, *H. vulgare* and *H. spontaneum*) identified 30 544 wild barley genes in 19 709 families. Of these gene families, 10 682 were shared by all five genomes and 1298 were unique in wild barley (Figure 1a).

A high‐confidence phylogenetic tree of the nine species was constructed using genes extracted from 1902 single‐copy families (Figure 1b). As expected, the wild barley had the closest relationship with cultivated barley. The dN/dS value (the ratio of the rate of non‐synonymous substitution to the rate of synonymous substitution) of the wild barley lineage was the highest among the compared species.

The Ks distribution of the one‐to‐one orthologous pairs of WB1‐*B. distachyon*, WB1‐*T. urartu*, WB1‐*S. talica*, WB1‐*S. bicolor*, WB1‐*O. sativa* and WB1‐*Z. mays* suggested the different divergence time between wild barley and other grass genomes (Figure 1c), which was consistent with the phylogenetic relationship generated by MrBayes analysis. The estimated divergence time between WB1 and *T. urartu*, *B. distachyon*, *S. talica*, *S. bicolor, O. sativa* and *Z. mays* was approximately 12.5, 32.6, 51.8, 54.9, 57.5 and 64.8 MYA, respectively (Figure 1d).

CAFÉ analysis finds that 1692 gene families were expanded and 2427 gene families were contracted in the wild barley genome compared with other species during the evolution. Compared to cultivated barley, the total number of significantly expanded families (*P* < 0.001) in WB1 were much higher (541 for WB1 compared with 107 for Morex), while the significantly contracted (*P* < 0.001) families in WB1 (92) were far less than those in Morex (373). The significantly expanded families in wild barley included many functional domains involved in plant stress response like F‐box domain and wound‐inducible basic protein family, as well as functional domains involved in reproductive process (Table S8). The significantly contracted families in wild barley included functional domains involved in protein synthesis, disease resistance and photosynthesis system (Table S9).

### The syntenic relationship between WB1 and Morex genome

**Figure 2:**
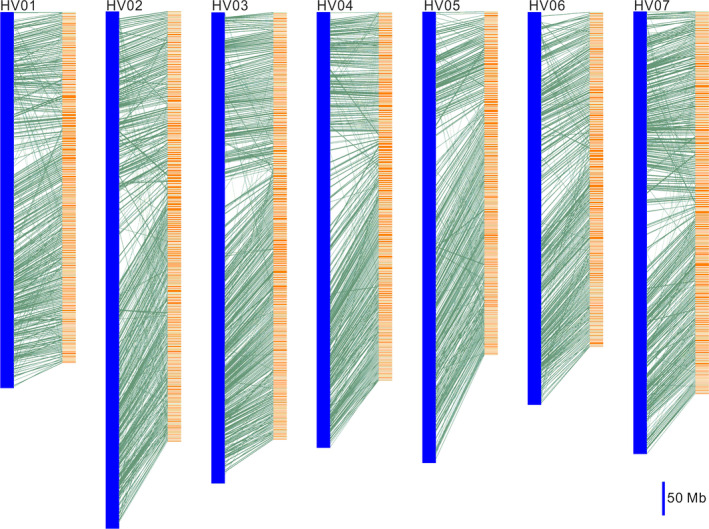
Gene synteny between wild barley and cultivated barley (Morex). The Morex chromosomes are represented by blue blocks (e.g., HVU01). The wild barley scaffolds (length >500 kb) are represented by orange blocks. Aligned genes are connected by green lines. The lengths of the chromosomes and scaffolds are shown relative to a 50‐Mb scale bar.

A total of 17 724 genes located on scaffolds with length over 500 kb in WB1 were aligned to the Morex gene models. This alignment identified 1558 syntenic blocks consisting of 9718 genes between the two genomes, and an average of six genes was found for each syntenic block (Table S10). The large number of syntenic blocks suggested good gene collinearity between the genomes of WB1 and Morex (Figure 2).

### Enrichment in genes related to resistance and tolerance to biotic and abiotic stresses in the genome of the wild barley

**Figure 3:**
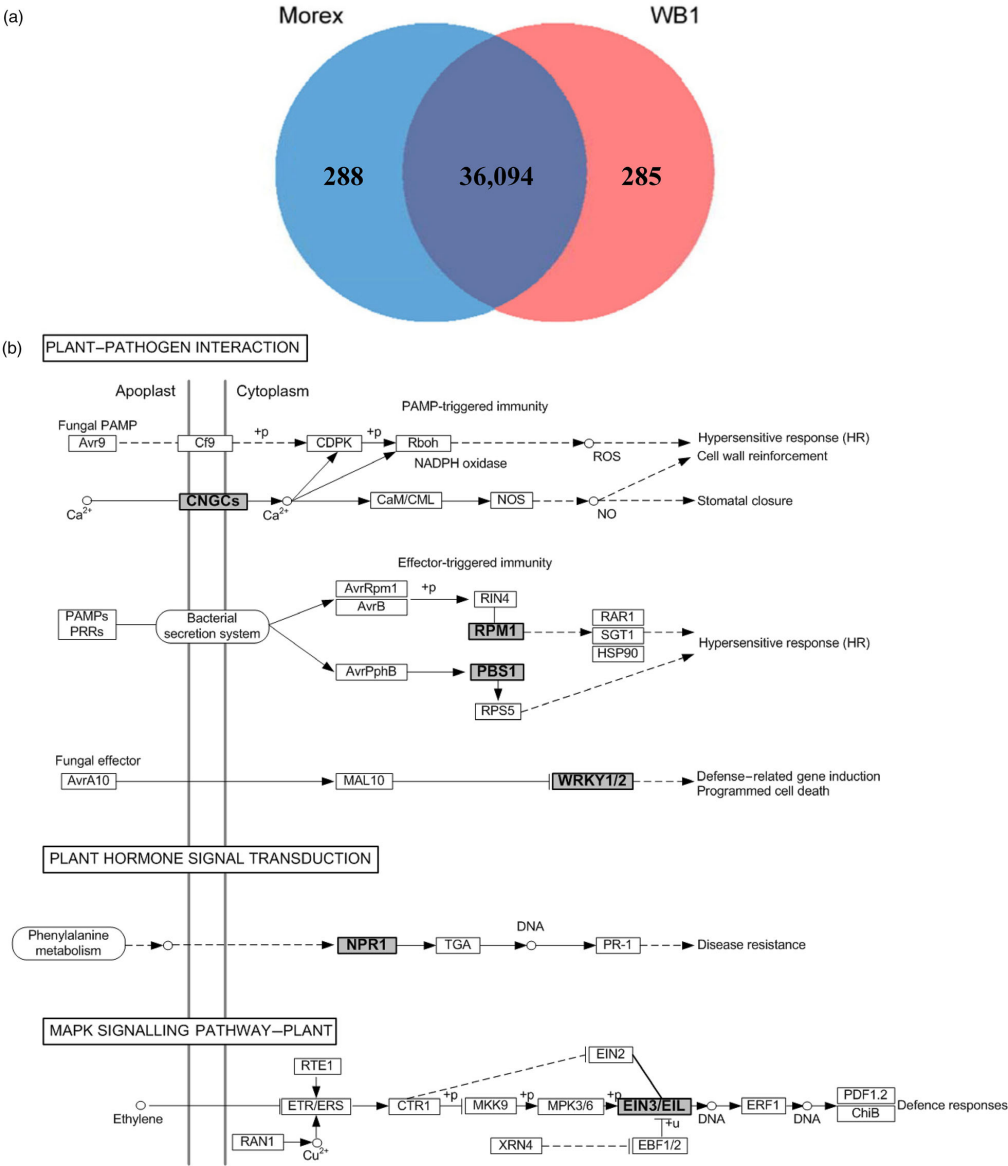
Genes specific to the wild barley genotype WB1. (a) Comparison with Morex genome and genes. (b) The enriched KEGG pathways and matched immunity‐related genes (in bold and with grey background) for wild barley‐specific genes.

A total of 569 WB1 genes were not found in Morex even at the settings of the minimum identity at 80% and coverage as 50%. Of these, 73 were syntenous with those in Morex and were excluded. After read mapping against Morex sequences, 285 of them were identified to be high‐confidence genes specific in WB1 (Figure 3a and Table S11). A similar analysis found 288 high‐confidence genes specific in Morex (Figure 3a, Table S12).

When analysed against the KEGG pathway database (Table S13), the WB1‐specific genes were found to be enriched in pathways of plant–pathogen interaction, plant hormone signal transduction and MAPK signalling (Figure 3b). These pathways are all associated with hypersensitive response (HR), disease resistance or defence responses. In particular, genes in the following six families related to biotic and abiotic stresses are among them: (i) the calmodulin binding protein CNGCs (cyclic nucleotide‐gated ion channels) are involved in plant immunity in *A. thaliana* (Moeder *et al.*, 2011; Schuurink *et al.*, 1998); (ii) the *Arabidopsis* gene RPM1 (resistance to *Pseudomonas maculicola* 1) (AT3G07040) is functionally known as an NBS‐LRR gene and confers resistance through a HR (Mackey *et al.*, 2002); (iii) the gene of PBS1 was an important PAMP‐triggered immunity signalling component (Sun *et al.*, 2017); (iv) WRKY proteins act as repressors of PAMP‐triggered basal defence (Shen *et al.*, 2007); (v) NPR1 (non‐expressor of PR genes) was associated with resistance to *Fusarium* head blight in wheat (Diethelm *et al.*, 2014; Yu *et al.*, 2017); and (vi) *Ta*EIL1k is a wheat homologue of *At*EIN3, which acts as a negative regulator, and its suppression could enhance the resistance of the wheat–stripe rust fungus interaction (Duan *et al.*, 2013). The Morex‐specific genes were significantly enriched in pathways of biosynthesis of secondary metabolites, photosynthesis, biosynthesis of amino acids and carbon metabolism (Table S13).

The genes specific to WB1 and Morex were also analysed with OrthoMCL. This analysis found that significantly enriched GO terms for WB1‐specific genes are involved in ‘DNA integration’, ‘DNA metabolic process’, ‘nucleic acid metabolic process’ and ‘response to wounding’. Significantly enriched top GO terms for Morex‐specific genes are mostly involved with ‘organonitrogen compound metabolic process’, ‘photosynthesis’, ‘gene expression’ and ‘biosynthetic process’. Significantly enriched GO terms of biological progress for the significantly expanded genes in WB1 (3149 genes) are involved with ‘telomere maintenance/organization’, ‘response to DNA damage stimulus’, ‘DNA metabolic process’ and ‘DNA repair’. For those expanded genes in Morex (806 genes), significantly enriched GO terms mainly include ‘defence response’, ‘response to stress and stimulus’, ‘carbon fixation’, ‘ATP synthesis coupled’ and ‘organonitrogen compound metabolic process’. Significantly enriched GO terms for singletons in the WB1 genome (8697 genes) mainly include ‘regulation of metabolic process’, ‘regulation of DNA replication’, ‘response to biotic stimulus’ and ‘defence response’. For those singletons in the Morex genome (9134 genes), GO terms of ‘response to auxin’, ‘oxidation‐reduction process’, ‘photosynthetic electron transport in photosystem II’ and ‘response to oxidative stress’ are significantly enriched (Table S14).

**Figure 4:**
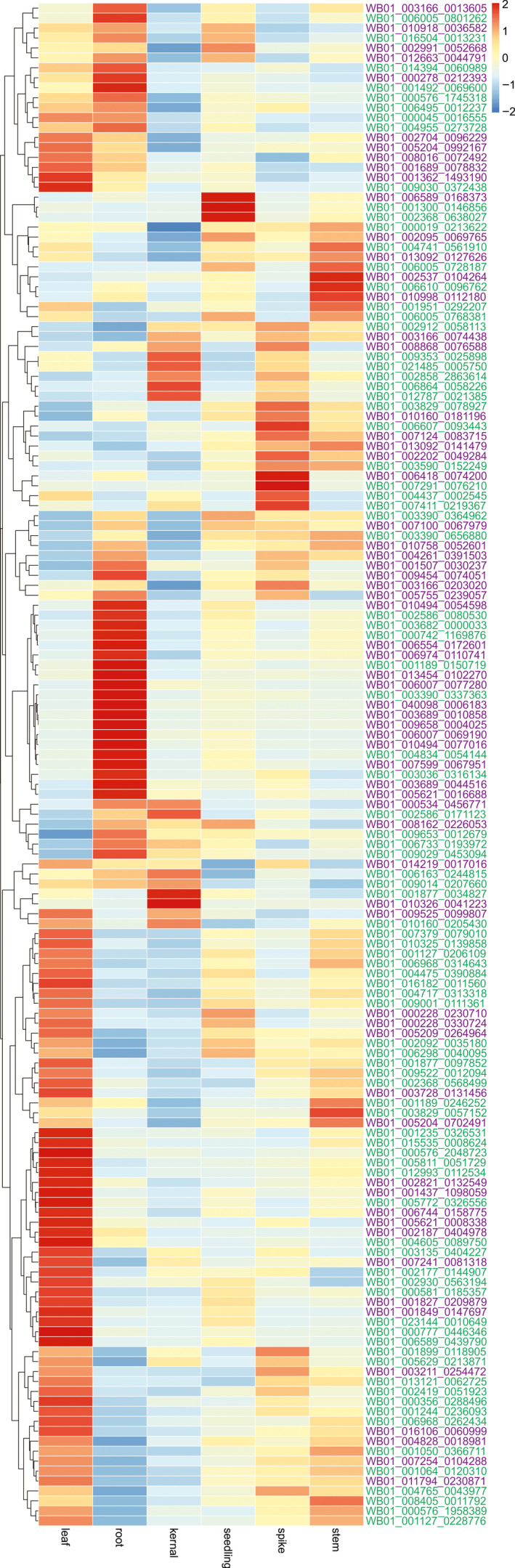
Expression patterns of NBS‐LRR genes in different WB1 tissues.

Based on the Pfam annotation analysis, there is no significant difference in the total count of NB‐ARC domain‐containing proteins between WB1 and Morex (high‐confidence gene sets only) (Table 2). However, WB1 has more genes with both NB‐ARC and LRR domains (271) than those of Morex (241). In addition, WB1 possesses a significantly higher number of complete CC‐NBS‐LRR genes (196) than Morex (128). The phylogenetic tree based on 512 NBS‐LRR genes (271 genes of WB1 and 241 genes of Morex) had divided those proteins into three main groups: (i) including 15 Morex genes and only one WB1 genes (highlighted in red colour); (ii) including 94 Morex genes and 113 WB1 genes (highlighted in blue colour); and (iii) including 133 Morex genes and 157 WB1 genes (highlighted in green colour) (Figure S9). Of the NBS‐LRR genes in WB1, 153 were highly expressed in different tissues. Differential expression patterns for these genes were observed across the tissues with most of them showing highest expression in root and leaf (Figure 4). Several of these genes were highly expressed in more than one tissue.

**Table 2:** Difference in the number of gene families related to stress tolerance and disease resistance between WB1 and Morex

Protein domain (Pfam and BLASTP analysis)	Number of genes	
	WB1	Morex
AP2/ERF	171	153
NAC	136	135
WRKY	100	113
MYB	290	281
ADH	140	144
B3	203	139
bZIP	92	89
HLH	144	133
LEA	133	120
NBS	436	418
TIR‐NBS	3	5
RPW8‐NBS	0	6
NBS‐LRR	271	241
TIR‐NBS‐LRR	0	0
RPW8‐NBS‐LRR	0	4
CC‐NBS‐LRR	196	127

Besides, we also found more stress‐related genes in WB1 (1411) than in Morex (1295), especially for gene families with B3 DNA binding domain, HLH domain and LEA protein domains (Table 2). The larger number of ERF and MYB gene families in WB1 is also consistent with the TF analysis (Figure S8).

### High levels of genetic variation in the pericentromeric regions were detected between the genomes of the wild and cultivated barley

**Figure 5:**
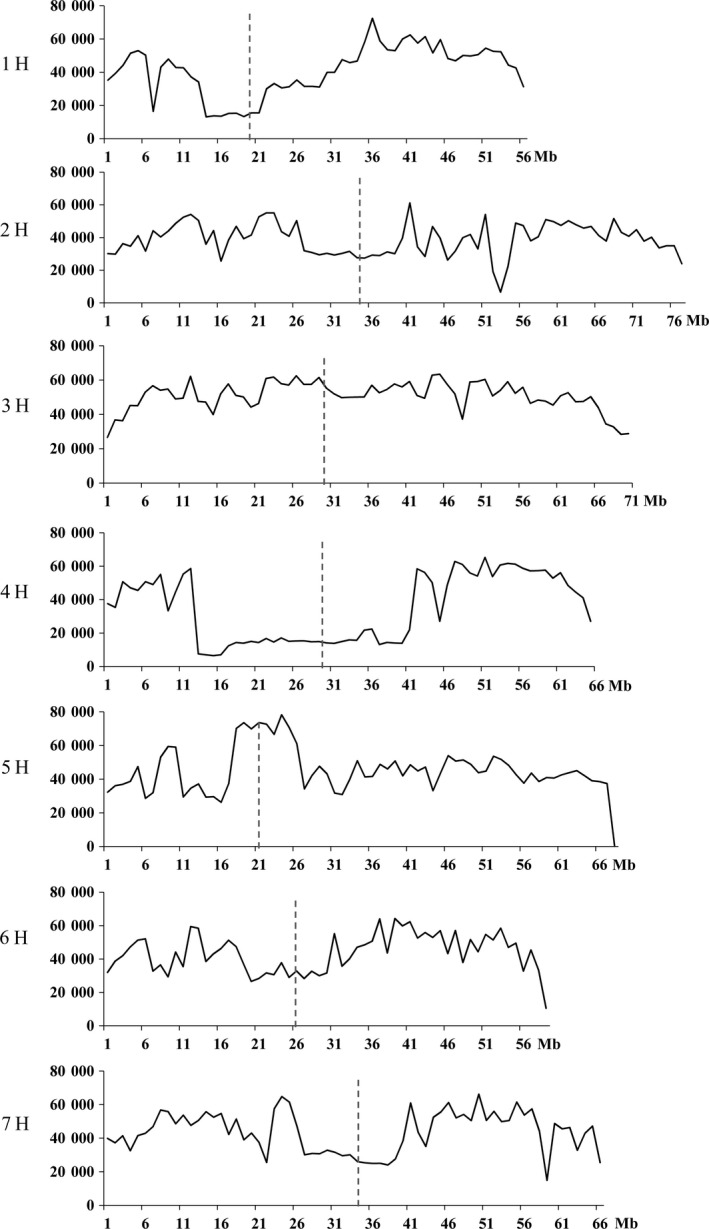
Distribution of SNV density (counts per 10 Mb interval) across the seven chromosomes of Morex. Interval length is 10 Mb for *x*‐axis. The vertical black dotted lines indicate the approximate locations of centromeres.

Distributions of the 19 649 075 SNVs detected between WB1 and Morex varied across different chromosomes. Chromosome 3H had the highest number (3 553 729), while 4H the lowest (2 259 870). Interestingly, the significantly lower density of variation commonly observed in the pericentromeric regions was not found in this analysis (Figure 5). Apart from those on chromosomes 1H and 4H, high SNV densities in the pericentromeric regions were detected for all other chromosomes especially for 3H and 5H (Figure 5). Thus, the wild barley genotype can be very effective in enhancing diversity for genes in these regions in breeding programmes. Genes with SNVs in the pericentromere region on chromosome 3H (220–400 Mb) are mainly involved in ‘ADP binding’, ‘oxidation‐reduction process’, ‘metal ion binding’, ‘mitochondrial matrix’ and ‘tRNA‐intron endonuclease activity’. Genes with SNVs in the pericentromere region on chromosome 5H (180–270 Mb) are mostly associated with ‘ATP/protein/DNA/RNA binding’, ‘oxidation‐reduction process’, ‘metal ion binding’, ‘response to stress’ and ‘oxidation‐reduction process’.

## Discussion

Until now, a whole‐genome assembly of wild barley is not available. To facilitate the exploitation of the abundant genetic variations in this taxon, we have generated a high‐quality draft genome assembly with 4.28 Gb in length and containing 36 395 high‐confidence protein‐coding genes. The assessment against the BUSCOs and barley fl‐cDNAs alignment showed that the gene‐containing regions have been well assembled and that the completeness of the WB1 genome is better than some other plant species. As anticipated, the genomes of the wild and cultivated barley are highly syntenic, with only a few misalignments. The genome size of WB1 is slightly smaller than that of Morex, which may be related to the difference in LTR content. The estimated distributions of divergence rates and LTR insertion times are comparable to those reported earlier, showing that wild and cultivated barley were differentiated about 10 000 years ago (Badr *et al.*, 2000; Salamini *et al.*, 2002; Zohary and Hopf, 2000).

Likely as a result of adapting to hostile environments, wild barley is known to have better tolerance to biotic and abiotic stresses than cultivated genotypes (Nevo, 2013; Nevo and Chen, 2010; Wang *et al.*, 2018) . Our results show that, compared with other species, about one third more gene families have been expanded in WB1 in comparison with those contracted gene families since the divergence of the wild barley with other species during evolution. In particular, we found that the number of significantly expanded families with functional domains involved in stress response in wild barley was significantly (*P* < 0.001) higher than those in Morex. Similar results were obtained by comparing the pan‐transcriptomes between the wild and cultivated barley genotypes (Ma *et al.*, 2019). Considering that kernel yield is one of the main improvements during domestication (Horns and Hood, 2012), these differences support the notion that resistance and tolerance can incur costs in yield potential (Horns and Hood, 2012; Tian *et al.*, 2003). Thus, targeting only those genes for major biotic and abiotic threats for a given environment can be more efficient than trying to incorporate all genes known to confer resistance into a single genotype when breeding for high‐yield varieties.

The numbers of high‐confidence genes specific to WB1 and Morex detected in this study were 285 and 288, respectively. These numbers were higher than those between Morex and the hulless genotype Zangqing320 (Dai *et al.*, 2018), indicating a closer genetic relationship between Morex and Zangqing320. The presence of these genotype‐specific genes was also anticipated as the existence of variable genomes has become clear in recent years (Gordon *et al.*, 2017; Li *et al.*, 2014; Lu *et al.*, 2015; Zhao *et al.*, 2018). Similar to those in the whole‐genome assembly, many of the WB1‐specific genes are related to disease resistance or defence responses, while the Morex‐specific genes were more enriched in pathways involved in growth and development‐related biological processes. Clearly, the potential of exploiting the wild barley taxon in breeding does not limit to the enrichment of alleles for those genes already present in the cultigen but can also allow the incorporation of novel genes lost during domestication.

A ‘V‐shaped’ distribution of the genetic diversity was normally observed among wild or cultivated barley groups (Baker *et al.*, 2014; Ma *et al.*, 2019; Russell *et al.*, 2016; The International Barley Sequencing Consortium, 2012). This, however, was not the case for most of the chromosomes for the variation detected between WB1 and Morex. Instead of giving the highest level of diversity towards the ends for any chromosome, a drop‐in SNV frequency was detected for most of the chromosomes in this study. Similar results were also obtained by comparing the hulless barley genotype Zangqing320 with cultivated ones (Dai *et al.*, 2018), who found that the density of SNVs in the centromeric and pericentromeric regions was not significantly lower than that in other regions in some of the chromosomes. The different results indicate that the effects of domestication on genetic diversity along chromosomes may not be even. Higher levels of genetic diversity were retained for the pericentromeric and telomeric regions. These results suggest that exploiting wild barley taxon may also be effective in enhancing diversity for genes in these regions in breeding programmes.

## Methods

### Plant materials and DNA sequencing

The wild barley genotype WB1 was used for sequencing in this study. There were two main reasons for the selection of this genotype. Firstly, it was collected from a site near the centre of barley origin; and secondly, it shows strong resistance to Fusarium crown rot (Chen *et al.*, 2013). The genotype was purified by six rounds of single‐seed descent to eliminate heterogeneity and reduce residual heterozygosity. Several plants derived from a single seed were then used for the study reported here. Differences in morphology between these plants and those from seeds provided by the Australian Winter Cereals Collection were not observed. The plants were grown in a glasshouse under a 24 °C day/20 °C night temperature cycle. High molecular weight genomic DNA was isolated from fresh leaves of a single plant using a standard cetyltrimethylammonium bromide extraction (Stein *et al.*, 2001).

About 4 μg genomic DNA was used in constructing paired‐end sequencing libraries. They were prepared with average insert sizes of 400 bp using paired‐end kits (Illumina, San Diego, CA) and sequenced by Illumina platforms (HiSeq 2500) with read length of 150 bp (Table S1). For construction of mate‐paired sequencing libraries, about 40 μg genomic DNA was used. The average insert sizes used for these libraries were 2.5, 9.0 and 13.0 kb, respectively. The libraries were constructed using Illumina Nextera mate‐pair kits and sequenced with 100 bp paired‐end reads (Table S1). Library preparation, sequencing and base calling were performed according to the manufacturer’s protocol (Illumina) by the Australian Genome Research Facility Ltd (AGRF, Parkville, Vic., Australia).

### Generation of Illumina RNA‐seq data

Six different tissues were used for generating RNA‐seq data: (i) whole seedlings of 15 days after planting (dap); (ii) leaves from seedlings at 25 dap; (iii) roots collected at 30 dap; (iv) first stems dissected at 42 dap; (v) spikelets obtained at anthesis; and (vi) developing kernels collected at 15 days post‐anthesis. Total RNA was extracted from frozen samples using the RNeasy Plant Mini Kit (Qiagen, Hilden, Germany). About 10 µg RNA from each of the tissues was used for sequencing based on Illumina HiSeq 2000 to produce 100 bp paired‐end reads (Table S2). All sequencing was done by AGRF Australia.

### Genome size estimation

For estimating the genome size of WB1, young leaves were collected from the plants of WB1 and maize and used for flow cytometric analysis. The mixed samples of WB1 and maize were assessed by flow cytometry with ten replications. The standard deviation was estimated by Excel. The term *C*‐value refers to the amount (picograms) of DNA contained within a haploid nucleus or one half the amounts in a diploid somatic cell of a eukaryotic organism. The software Summit V5.2.0.7477 (http://www.cyto.purdue.edu/cdroms/cyto5/sponsors/cytomate/summit.htm) was adopted to analyse the results. Kmerfreq_AR (SOAPec_v2.01 package https://sourceforge.net/projects/soapdenovo2/files/ErrorCorrection/SOAPec_v2.01.tar.gz/download) was also used to estimate the genome sizes of WB1 and Morex. The raw data of Morex used for Kmerfreq_AR were downloaded from the website https://www.ebi.ac.uk/ena/data/view/PRJEB3027.

### Genome assembly

With hierarchical libraries of different insertion sizes, a combined strategy was employed to assemble the whole genome (Figure S1). SOAPdenovo2 (Luo *et al.*, 2012) (http://soap.genomics.org.cn/soapdenovo.html) was adopted to assemble the paired‐end reads into contigs (at k‐mer size 95). Scaffolds were built with the use of mate‐pair reads (at k‐mer size 55), and other parameters at default values. Gapcloser (https://sourceforge.net/projects/soapdenovo2/files/GapCloser/) were then used to fill sequence gaps with default parameters. At the same time, the software Fermi (Li, 2012) (http://github.com/lh3/fermi) was used to assemble the paired‐end reads, which implemented a string graph algorithm. The result from Fermi was then merged to the scaffolds from SOAPdenovo2 to fill sequence gaps.

### Quality assessment of the assembled wild barley genome

The 956 single‐copy plant genes in the BUSCO (Simão *et al.*, 2015) gene set were used to evaluate the completeness of the wild barley genome assembly. The fractions of missing and fragmented genes indicate the degree of incompleteness of the genome assembly and gene prediction. As all genes in the BUSCO set are single‐copy ones, the presence of duplicates would suggest the erroneous assembly of the haplotypes.

The quality of the genome assembly was also evaluated in the following aspect: A total of 28 620 publicly available full‐length cDNAs (fl‐cDNA) from *H. vulgare* were collected from the NCBI database: accession numbers AK248134 to AK253139 for 5006 of them (Sato *et al.*, 2009) and AK353559 to AK377171 for the remainder 23 614 (henceforth, the National Institute of Agrobiological Sciences FLcDNAs and NIAS FLcDNAs). These 28 620 fl‐cDNAs were clustered into non‐redundant sequences using CD‐Hit (Li and Godzik, 2006) at 98% identity level. At last, all non‐redundant barley fl‐cDNAs were aligned to the assembled wild barley genome using GMAP (Wu and Watanabe, 2005).

To check the level of organelle contamination, the genome assembly of WB1 was aligned against the sequences of *H. vulgare* chloroplast (Saski *et al.*, 2007) and mitochondrial DNA of *H. vulgare* and *H. spontaneum* (Hisano *et al.*, 2016) with a total size of ~1.13 Mb using the software MUMmer3.22 (Kurtz *et al.*, 2004) with parameters of ‘show‐coords –rcl, delta‐filter –q, and show‐coords ‐qcl’. The total length of matched sequences was 783 287 bp, indicating that the contamination proportion of WB1 genome assembly was approximately 0.02%.

### Annotation of repeat elements

The *ab initio* prediction program RepeatModeler (http://www.repeatmasker.org/RepeatModeler.html, version 1.0.5) was employed to build the *de novo* repeat library in the assembled genome using the default parameters. The yielded consensus sequences were then manually checked by aligning to genes from the NCBI database (nt and nr; released June 2013) to remove multicopy gene sequences. Using the library consisting of 510 consensus sequences as a database, RepeatMasker (http://www.repeatmasker.org/, version 3.3.0) was implemented to identify and classify homologous repeat elements in the genome. The repeat annotations of the cultivated barley genotype Morex, rice and maize were analysed using the same pipeline. The LTR_FINDER program (Xu and Wang, 2007) was used to identify the full‐length LTR (full‐length long terminal repeat) retrotransposons in the WB1 and Morex genomes based on the default settings. The two LTRs were aligned, and the *K* value (the average number of substitutions per aligned site) was calculated with MEGA7 (Kumar *et al.*, 2016). The insertion times were estimated using the formula: *T* = *K*/(2 × *r*), where *r* represents the average substitution rate and is estimated to be 1.3 × 10^−8^ substitutions per site per year (Ma and Bennetzen, 2004).

### Gene model prediction and functional annotation

Gene model prediction was based on the integration of *de novo* gene prediction and evidence‐based prediction, which included sequence homology‐based predictions and RNA‐seq data mapping. All predicted gene structures were integrated into consensus gene structures using EVM annotation pipeline (Haas *et al.*, 2008). *De novo* prediction was carried out using the prediction software tools Augustus (Stanke *et al.*, 2006) trained on the wheat training set and FgeneSH++ (http://www.softberry.com) with gene model parameters trained from monocots to build the preliminary gene models on repeat‐masked genome sequence. Protein‐based homology searches and intron resolution were conducted using the Exonerate software (Slater and Birney, 2005) against the protein sequences of *A. thaliana, S. bicolor, Z. mays, S. talica, A. tauschii, Triticum aestivum, T. urartu*, *H. vulgare*, *B. distachyon* and *O. sativa* downloaded from Gramene (http://www.gramene.org/). The RNA‐seq reads were aligned against the repeat‐masked genome sequence using TopHat2 (Trapnell *et al.*, 2012) with default parameters. The resulting alignment files were then assembled into transcript structures using genome‐guided assemblers Cufflinks (Trapnell *et al.*, 2012). RNA‐seq data were also assembled using de novo assemblers Trinity (Grabherr *et al.*, 2011) with the default parameters. PASA (Campbell *et al.*, 2006) was used to reassemble the transcripts based on overlapping alignments from full‐length cDNAs and RNA‐seq assemblies. All the evidences from above (including *de novo* prediction, protein‐homology searches and RNA‐seq assemblies) were merged to form a comprehensive and consensus gene set by EVM. Two strategies were carried out to refine the preliminary gene models: Firstly, all the predicted gene models were compared with rice and sorghum gene model sets by BLASTP with an *E*‐value cut‐off of 1.0 × 10^−20^. Those models with a minimum coverage of 20% were retained. The coverage of each wild barley gene is the ratio of aligned protein sequence length to total protein length of the corresponding gene. Secondly, the Illumina RNA‐seq sequences from the six tissues (root, seedling, leaf, stem, developing kernel and spikelet) were mapped to the CDS using the SMALT aligner (http://www.sanger.ac.uk/resources/software/smalt/). The gene models with the RNA‐data coverage rate ≥50% and mapped reads depth ≥500 were retained. Completeness of gene‐space representation was evaluated with the BUSCO pipeline. The motifs and domains of genes were determined by InterProScan version 5.7 (Zdobnov and Apweiler, 2001) against protein databases including ProDom, PRINTS, Pfam, SMART, PANTHER and PROSITE. The functional ontology for each gene was retrieved from the outputs of InterPro using the Gene Ontology (Ashburner *et al.*, 2000).

### Transcriptome analyses

The Illumina RNA‐seq sequences from six different tissues (root, seedling, leaf, stem, developing kernel and spikelet) with two biological replicates were mapped to the assembled WB1 genome using hisat2 v2‐2.0.5 (Kim *et al.*, 2015). Normalized read counts based on the gene annotation and differential gene expression under various comparison schemes were identified using the R package DESeq2 (Love *et al.*, 2014). A gene with expression levels increased by twofold or higher (padj ≤ 0.01) in one tissue compared to the other five tissues was treated as tissue‐specific and highly expressed. All highly expressed genes were functionally annotated using agriGOv2.0 (Tian *et al.*, 2017), and the results were visualized using the R package ‘REVIGO’ with FDR‐corrected *P* < 0.05 (Supek *et al.*, 2011).

### Annotation of conserved non‐coding RNA genes

The miRNA, snRNA, C/D snoRNA and H/ACA snoRNA genes in the assembled wild barley genome were predicted by the cmsearch program of the INFERNAL software (Nawrocki and Eddy, 2013) against the Rfam database (release 11.0, 2207 families) (http://rfam.xfam.org/) with score cut‐off of 90 and *E*‐value cut‐off of 1e^−10^. tRNA genes were identified using tRNAscan‐SE‐1.3.1 with the default settings (Lowe and Eddy, 1997). In addition, tRNA genes from the two cultivated barley genotypes (Morex and *HarunaNijo*) and nine other plant species (*A. tauschii*, *T. urartu*, *T. aestivum, B. distachyon*, *A. thaliana*, *O. sativa*, *S. bicolor*, *Z. mays* and *S. italica*) were also predicted with the same procedure.

### Annotation of transcription factors

The PlantTFDB 4.0 (http://planttfdb.cbi.pku.edu.cn/index.php) was used to annotate all possible candidate TFs in the wild barley genome by retrieving the best hits in *A. thaliana*. For comparative analysis, the data of TF families of other related genomes were also downloaded from PlantTFDB.

### OrthoMCL gene family clustering

The genome and annotation data of *A. thaliana*, *S. bicolor*, *Z. mays, S. italica*, *A. tauschii*, *T. urartu*, *B. distachyon* and *O. sativa* from Gramene were downloaded from http://www.gramene.org/, and the genome and gene models of Morex were downloaded from http://dx.doi.org/10.5447/IPK/2016/34. Splice variants and the gene models with open reading frames shorter than 150 bp were removed to only keep the representative transcript for each gene model. After filtering, the pairwise sequence similarities between all input protein sequences were calculated using all‐by‐all BLASTP (Altschul *et al.*, 1990) with an *E*‐value of 1.0 × 10^−10^ and minimum match length of 50%. Gene family clustering was performed using OrthoMCL software version 2.0.9 (Li *et al.*, 2003) based on the high‐confidence protein‐coding genes of wild barley and the protein sets of the eight monocots and one dicot as mentioned above.

### Reconstruction of phylogeny and evolutional analysis of gene families among 9 fully sequenced plant genomes

Protein sequences from 1902 single‐copy orthologous gene clusters were extracted and used to construct the phylogenetic relationships among the nine species using MrBayes (Huelsenbeck and Ronquist, 2001). The GTR+gamma+I substitution model was used with the parameter set to 1 000 000 (1 sample/100 generations), and the first 2500 samples were burned in. Two independent runs were conducted using *A. thaliana* as an outgroup. Branch‐specific dN and dS were estimated with the CODEML program of PAML (Yang, 2007). We undertook a computational analysis of gene family sizes using the software CAFÉ 2.2 (De Bie *et al.*, 2006) to study gene family expansion and contraction during the evolution of wild barley and related species.

### Estimation of divergence time between wild barley and other grass species

Orthologous genes among wild barley, *T. urartu*, *B. distachyon*, *S. talica*, *S. bicolor, O. sativa* and *Z. mays*, were selected using the same method for the construction of gene synteny between WB1 and Morex. *K*s values were calculated using 1902 single‐copy gene clusters determined by the OrthoMCL pipeline and their orthologous genes in the syntenic blocks. The mean *K*s was used to estimate the divergence time between different genomes with the universal substitution rate of 6.5 × 10^−9^ mutations per site per year (Gaut *et al.*, 1996).

### Assessment of gene synteny between WB1 and Morex genomes

A pairwise co‐linearity analysis between the WB1 scaffolds with length over 500 kb and Morex chromosomes was conducted. Homologous gene pairs of WB1 and Morex were identified by all‐against‐all BLASTP with *E*‐value at 1.0 × 10^−10^. Two criteria were used to call syntenic gene blocks in the wild barley scaffolds: (i) number of the genes in one syntenic block ≥3, and (ii) number of non‐syntenic genes between two adjacent syntenic genes <5. A perl script following manual check was applied to determine the syntenic blocks.

### Identification of genes specific in the WB1 or Morex genome

To identify genes specific for either WB1 or Morex, cDNA of the newly assembled WB1 genes was aligned to the latest version of the published Morex genome sequences, and then, the cDNA of Morex high‐confidence genes was aligned to the WB1 genome using GMAP (Wu and Watanabe, 2005). The minimum identity and coverage were set at 80% and 50%, respectively (*E*‐value < 1 × 10^–5^). Collinear genes between the two genotypes were also excluded in identifying genes specific in either of the two genomes. To confirm those genes specific in the WB1 genome are truly missing from the Morex genome, raw sequences of Morex (Mascher *et al.*, 2017) were aligned to the WB1 genome with Bowtie2 (Langmead and Salzberg, 2012) and the read depth of each base position and the coverage profiles of these genes were counted using samtools (Li *et al.*, 2009). Those with more than 50% coverage in Morex were defined as high‐confidence genes specific in WB1. The same were done for identifying Morex‐specific genes. The KEGG’s (Kyoto Encyclopedia of Genes and Genomes) internal annotation tool BlastKOALA (www.kegg.jp/blastkoala/) and KAAS (www.genome.jp/tools/kaas/) at the GenomeNet mirror sites were used to assign K numbers to all of the final unique genes in the wild barley genome. KEGG mapping was then conducted using KEGG Mapper (www.kegg.jp/kegg/mapper.html) to identify the enrichment pathway.

OrthoMCL (Li *et al.*, 2003) analysis was carried out to define gene family clusters for the WB1 and Morex genomes. Over‐ and under‐representation of gene ontology (GO) terms in specific gene families and subsets were analysed by hypergeometric testing with agriGOv2.0 (Tian *et al.*, 2017). The redundant and similar terms from the long GO lists were removed by semantic clustering with Revigo (Supek *et al.*, 2011), and the final enrichment results were visualized (FDR‐corrected *P* < 0.05). Those specific groups include the following: (i) WB1‐ or Morex‐specific; (ii) singletons among the WB1‐ or Morex‐specific ones; and (iii) expanded genes in either WB1 or Morex, that is where relative gene copy numbers between them are significantly (>5‐fold, *P < 0.05*) expanded.

### Comparison of disease resistance and stress‐related gene families between WB1 and Morex

Proteins possessing NB‐ARC domains (Pfam: PF00931) from WB1 and the high‐confidence proteins of Morex were retrieved for analysing fused domains with HMMscan program from HMMER v3.1b2 software (Finn *et al.*, 2015) (*E*‐value < 1 × 10^–3^). Those additional NLR (nucleotide‐binding leucine‐rich repeat) domains are TIR (Toll/interleukin‐1receptor/resistance protein, PF01582), TIR2 (PF13676), LRRs (leucine‐rich repeats, CL0022) and RPW8 (PF05659) (McHale *et al.*, 2006). In addition, we scanned those proteins with NB‐ARC domains with NLR MEME motifs and analysed results with NLR‐parser to identify the CC (coiled‐coil) domain (Steuernagel *et al.*, 2015). As more than 80% of the cloned disease‐resistant genes belong to NBS‐LRR gene family (Liu *et al*., 2007), the NBS‐LRR genes of WB1 and Morex were selected for phylogenetic analysis with ete3 toolkit (Jaime *et al.*, 2016) and expression pattern analysis of WB1 NBS‐LRR genes in different tissues. Transcription levels were quantified with normalized read counts, and the heatmap was drawn using the R package. Stress‐related domains, including AP2/ERF (Xu *et al.*, 2011), NAC (Puranik *et al.*, 2012), WRKY (Jiang *et al.*, 2017), MYB (Ambawat *et al.*, 2013), ADH (Christie *et al.*, 1991), B3 DNA binding domain (Yamasaki *et al.,* 2004), bZIP (Hu *et al.*, 2016), HLH (Huang *et al.*, 2004) and LEA protein domain (Liu *et al.*, 2013), were also compared between the protein sets of WB1 and Morex using the Pfam annotation and BLASTP analysis.

### SNV identification by mapping sequences of WB1 to those of Morex

Following the removal of all low‐quality reads using SolexaQA++ software (Cox *et al.*, 2010), WB1 reads (150 bp paired‐end reads) were aligned to the Morex genome (Mascher *et al.*, 2017). SNVs (single nucleotide variations) were called using Biokanga v4.3.4 (Stephen *et al.*, 2012) with a minimum read depth of 4 and a maximum of two substitutions. To calculate the distribution of SNVs between the WB1 and Morex, a non‐overlapping window of 10 Mb along the Morex chromosome sequence was used to analyse the rates of SNVs. The location of centromere on each chromosome was predicted based on that of Morex (Mascher *et al.*, 2017).

### Availability of data

The draft genome sequence and annotation file of wild barley genotype WB1 (cv. AWCS276) are available at http://www.ncgr.ac.cn/wild_barley. All raw reads obtained in this work were deposited in the European Nucleotide Archive (ENA) with the accession number of PRJEB25923.

## Authors' contributions

CJL, BH and DCL conceived and designed the experiments. ML, YL, YLM, QZ, JS, QF and QLT conducted all other experiments and analysed the data. CJL, ML, YL, YLM and QZ wrote the paper. All authors read and approved the final manuscript.

## Conflict of interest

The authors declare that they have no competing interests.

## Supporting information


**Figure S1** Strategy used in assembling the wild barley genome.
**Figure S2** Estimation of the wild barley genome size by flow cytometry and kmerFreq‐AR.
**Figure S3** The distribution of sequence divergence rates of interspersed repeats in the genomes of the wild barley (WB1), cultivated barley (Morex), rice and maize.
**Figure S4** Comparison of LTR insertion times between the wild barley genotype WB1 and the cultivated genotype Morex.
**Figure S5** Statistics and functional classification of gene models identified from the wild barley genome.
**Figure S6** Numbers of tissue‐specific and highly expressed genes in six tissues.
**Figure S7** GO term enrichment of highly expressed genes in different tissues.
**Figure S8** Comparison of transcription factor among barley, other grass species and *Arabidopsis*.
**Figure S9** Phylogenetic analysis of 512 NBS‐LRR genes from WB1 and Morex.

**Table S1** Statistics of the whole genome shotgun sequences of WB1.
**Table S2** RNA‐seq raw reads of the wild barley genotype WB1.
**Table S3** *De novo* assembly of the wild barley genome.
**Table S4** BUSCO comparison between the wild barley and other published genomes.
**Table S5** Statistics of repetitive sequences in the wild barley genome.
**Table S6** A summary of tRNA genes identified in wild barley and its close relatives.
**Table S7** Families of non‐coding RNA (ncRNA) genes in the wild barley assembly.
**Table S10** Syntenic loci of the wild barley genotype WB1 on each of the chromosomes of Morex.

**Table S8** Significantly expanded gene families in the wild barley genome compared with other plant genomes.
**Table S9** Significantly contracted gene families in the wild barley genome compared with other plant genomes.
**Table S11** The Morex reads mapping coverage profiles for genes specific in WB1.
**Table S12** The WB1 reads mapping coverage profiles for genes specific in Morex.
**Table S13** A KEGG pathway summary for genes specific to Morex and WB1.
**Table S14** GO over‐repressentation in WB1 and Morex.

Supplementary File

Supplementary File

Supplementary File
