# Supplementary material for: The draft genome of a wild barley genotype reveals its enrichment in genes related to biotic and abiotic stresses compared to cultivated barley
Source: Plant Biotechnol J. 2019 Aug 4;18(2):443–56. doi: 10.1111/pbi.13210 (PMC6953193; doi:10.1111/pbi.13210)
Supplement: Supplementary file 1 — Figure S1 Strategy used in assembling the wild barley genome. Figure S2 Estimation of the wild barley genome size by flow cytometry and kmerFreq‐AR. Figure S3 The distribution of sequence divergence rates of interspersed repeats in the genomes of the wild barley (WB1), cultivated barley (Morex), rice and maize. Figure S4 Comparison of LTR insertion times between the wild barley genotype WB1 and the cultivated genotype Morex. Figure S5 Statistics and functional classification of gene models identified from the wild barley genome. Figure S6 Numbers of tissue‐specific and highly expressed genes in six tissues. Figure S7 GO term enrichment of highly expressed genes in different tissues. Figure S8 Comparison of transcription factor among barley, other grass species and Arabidopsis. Figure S9 Phylogenetic analysis of 512 NBS‐LRR genes from WB1 and Morex. [file PBI-18-443-s005.doc]

**Additional files**

**Additional file 1**


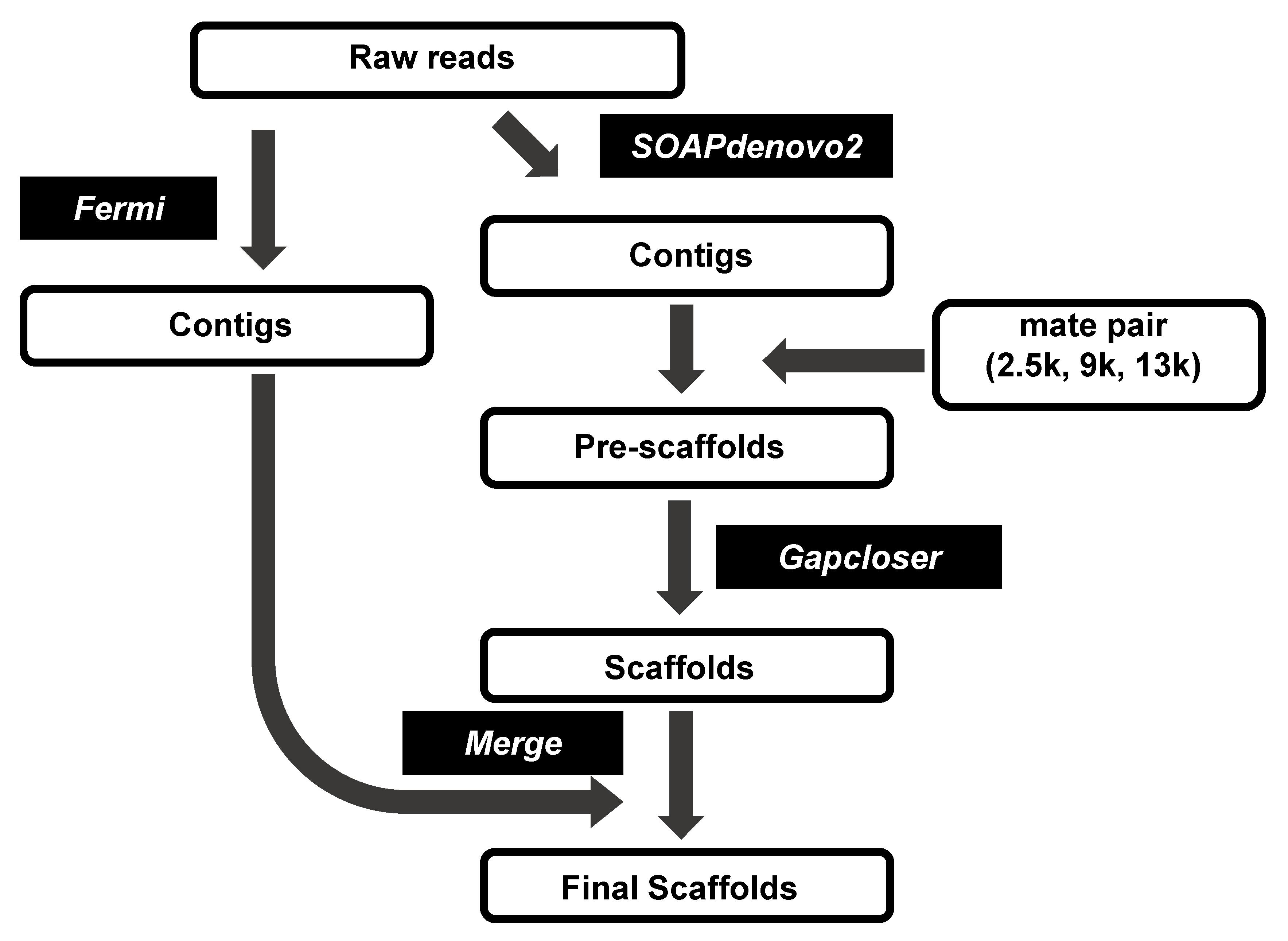


**Fig. S1. Strategy used in assembling the wild barley genome.** The raw reads were assembled by SOAPdenovo2 and Fermi, respectively. The contigs from Fermi were then merged to the scaffolds of SOAPdeonovo2 to fill more gaps.

A


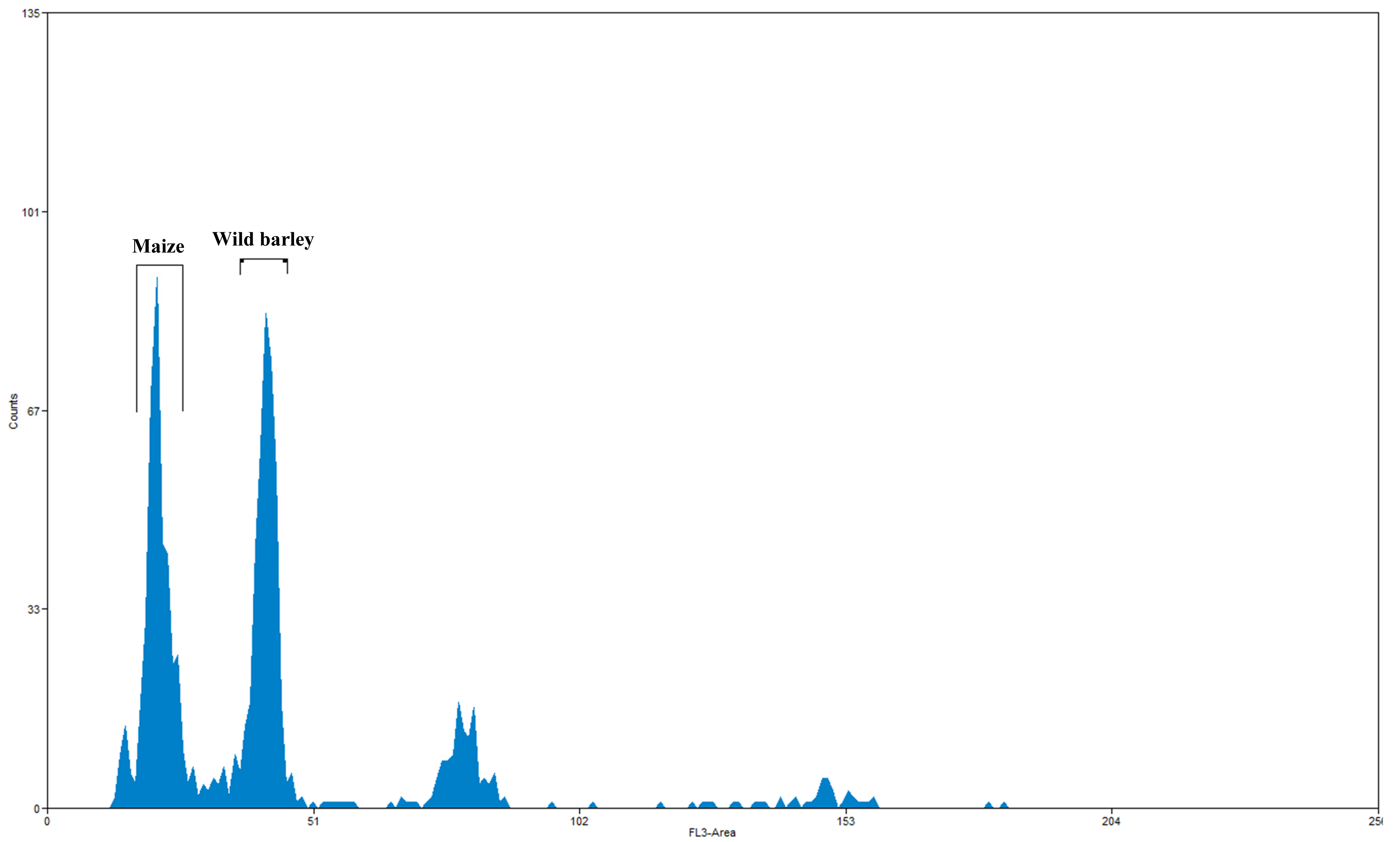


B

**
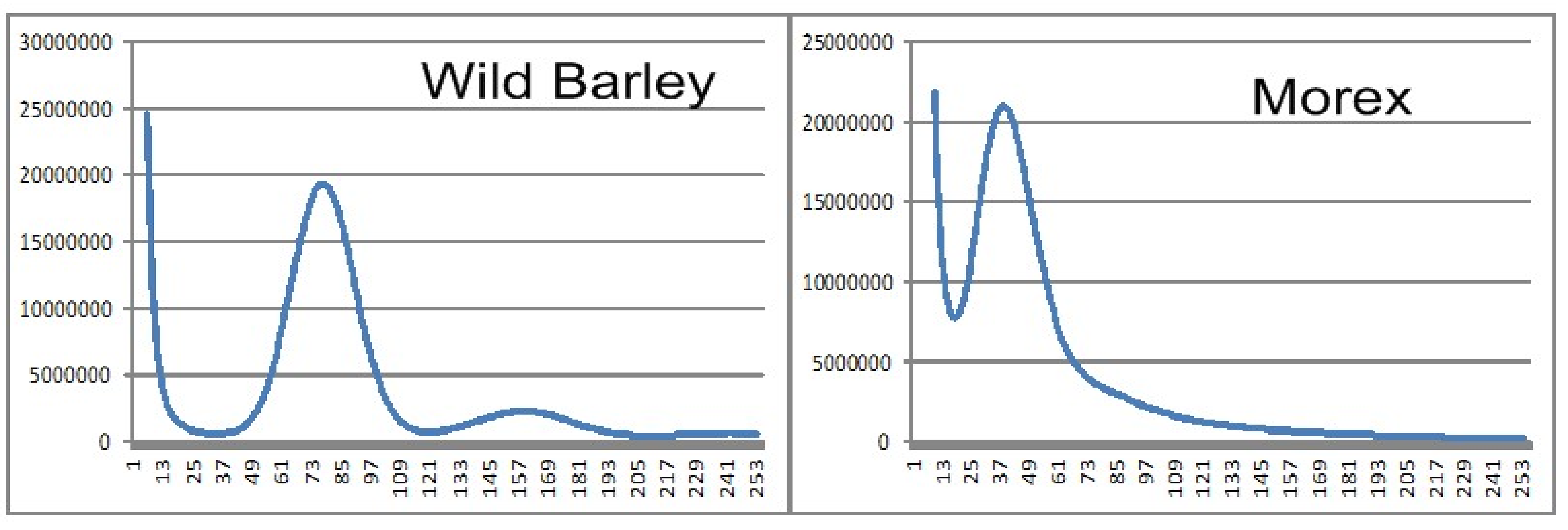
**

**Fig. S2. Estimation of the wild barley genome size by flow cytometry and kmerFreq-AR. (A)** Assessing mixed samples of WB1 and maize using flow cytometry. The term C-value refers to the amount (picograms) of DNA contained within a haploid nucleus or one half the amounts in a diploid somatic cell of a eukaryotic organism. The peak of maize indicated a 2 C DNA value for at 21.60, while the peak of wild barley indicated a 2 C DNA at 41.21. Compared with that of Maize (2.30 Gb), the genome size of wild barley was estimated to be ~4.60 Gb. (**B)** Distribution of Kmer frequency. Values of K-mers were plotted against the frequency (y-axis) at their occurrence (x-axis). The genome size of WB1 and Morex were estimated to be about 4.45 Gb and 4.76Gb respectively.

**
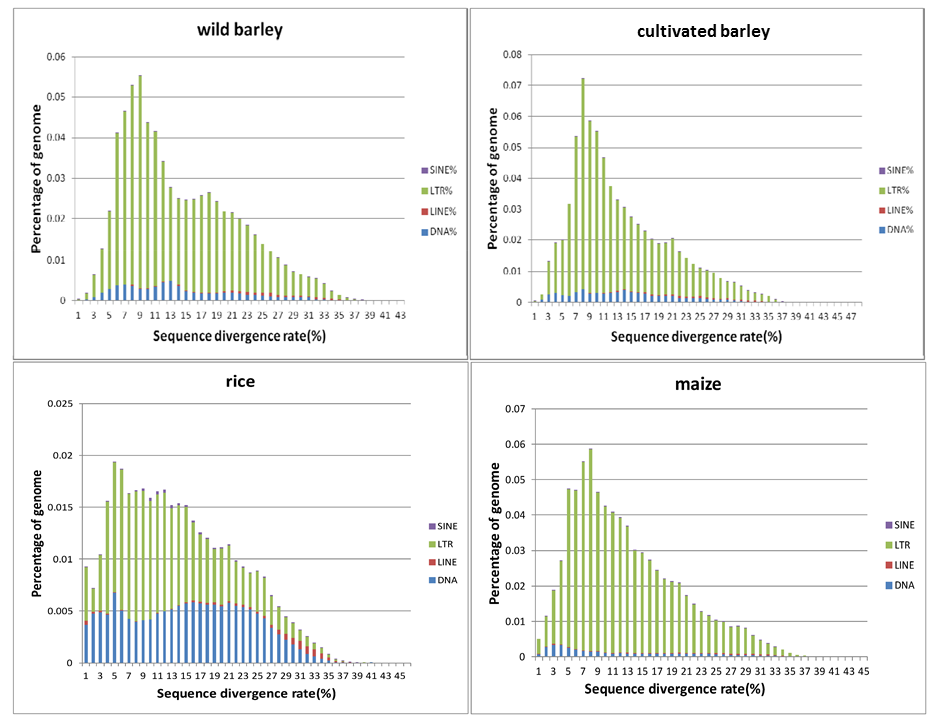
**

**Fig. S3. The distribution of sequence divergence rates of interspersed repeats in the genomes of the wild barley (WB1), cultivated barley (Morex), rice and maize.** The divergence rate was calculated based on the alignment between the RepeatMasker annotated repeat copies and the consensus sequence in the repeat library.


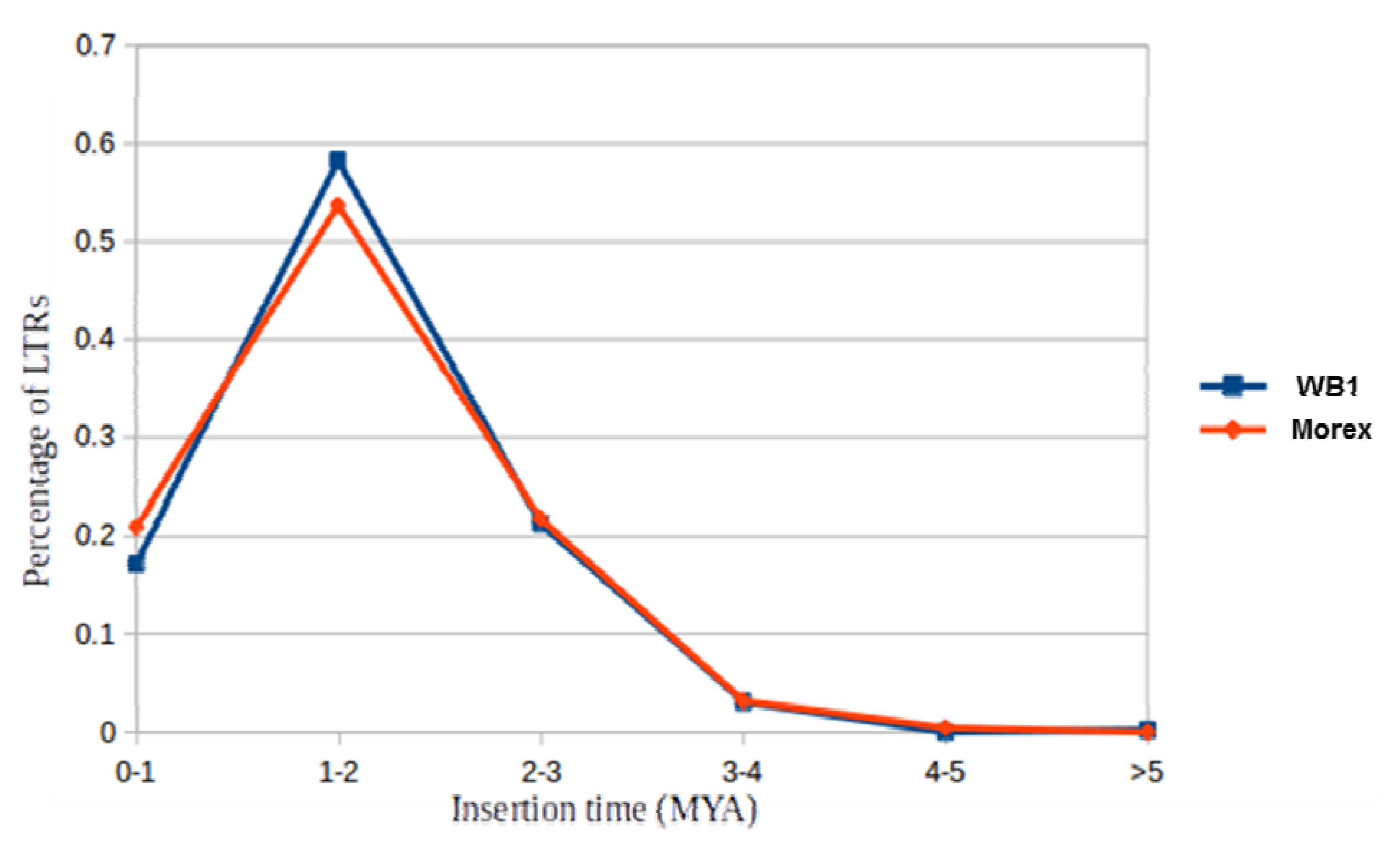


**Fig. S4. Comparison of LTR insertion times between the wild barley genotype WB1 and the cultivated genotype Morex.**


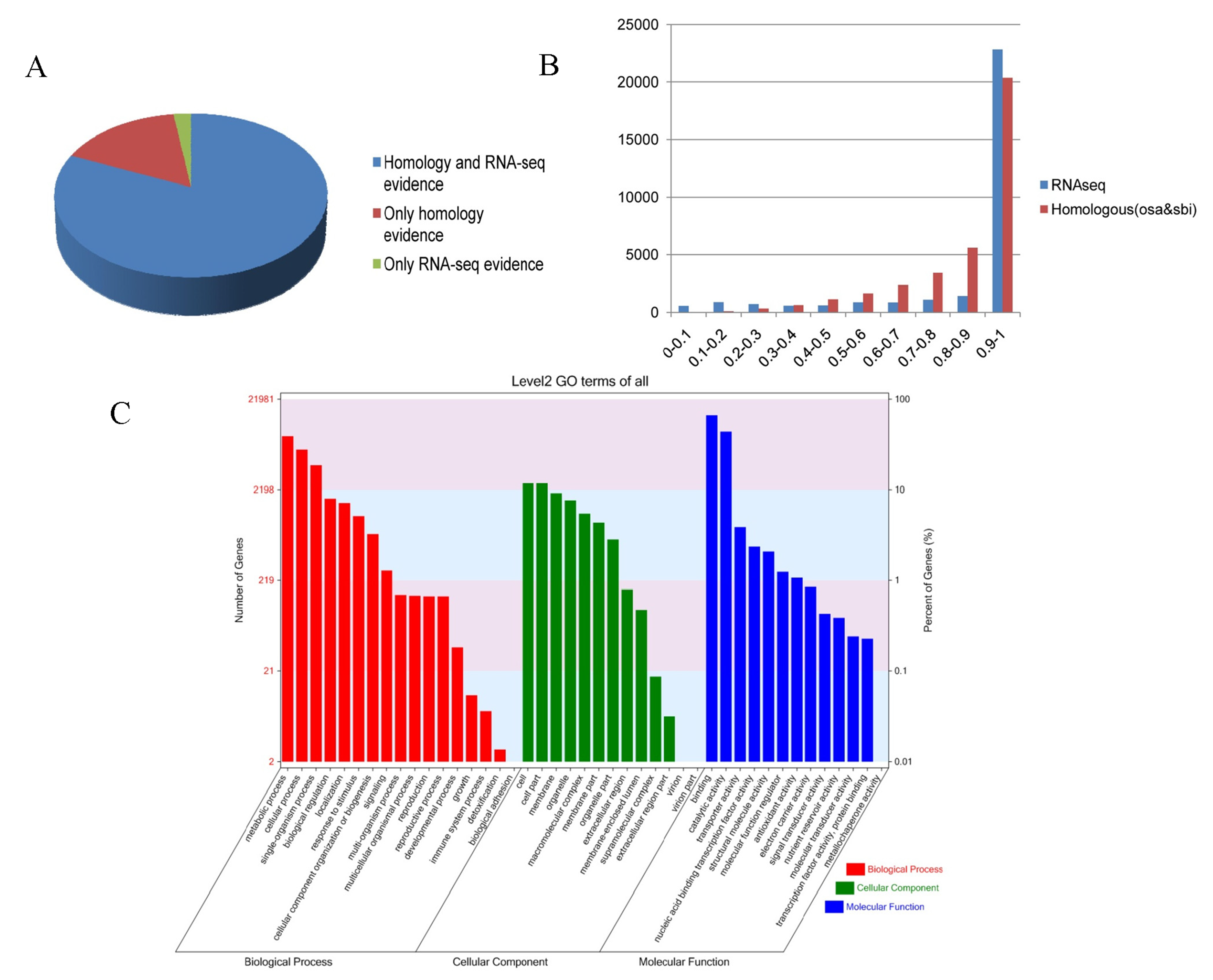


**Fig. S5. Statistics and functional classification of gene models identified from the wild barley genome. (A)** Gene models supported by evidences from protein-based homology searches and expression data. **(B)** The distribution of wild barley genes supported by homologous and RNA-seq expression evidence. The gene models of wild barley are aligned to those of rice and sorghum by BLASTP, the coverage for each of the wild barley genes is the ratio of aligned protein sequence length to total protein length of the corresponding gene. RNA-seq reads were mapped to gene coding sequences, and the coverage is the ratio of total aligned reads length to the coding sequence length of the gene in concern. **(C)** Functional classification of the wild barley gene models based on Gene Ontology Consortium.


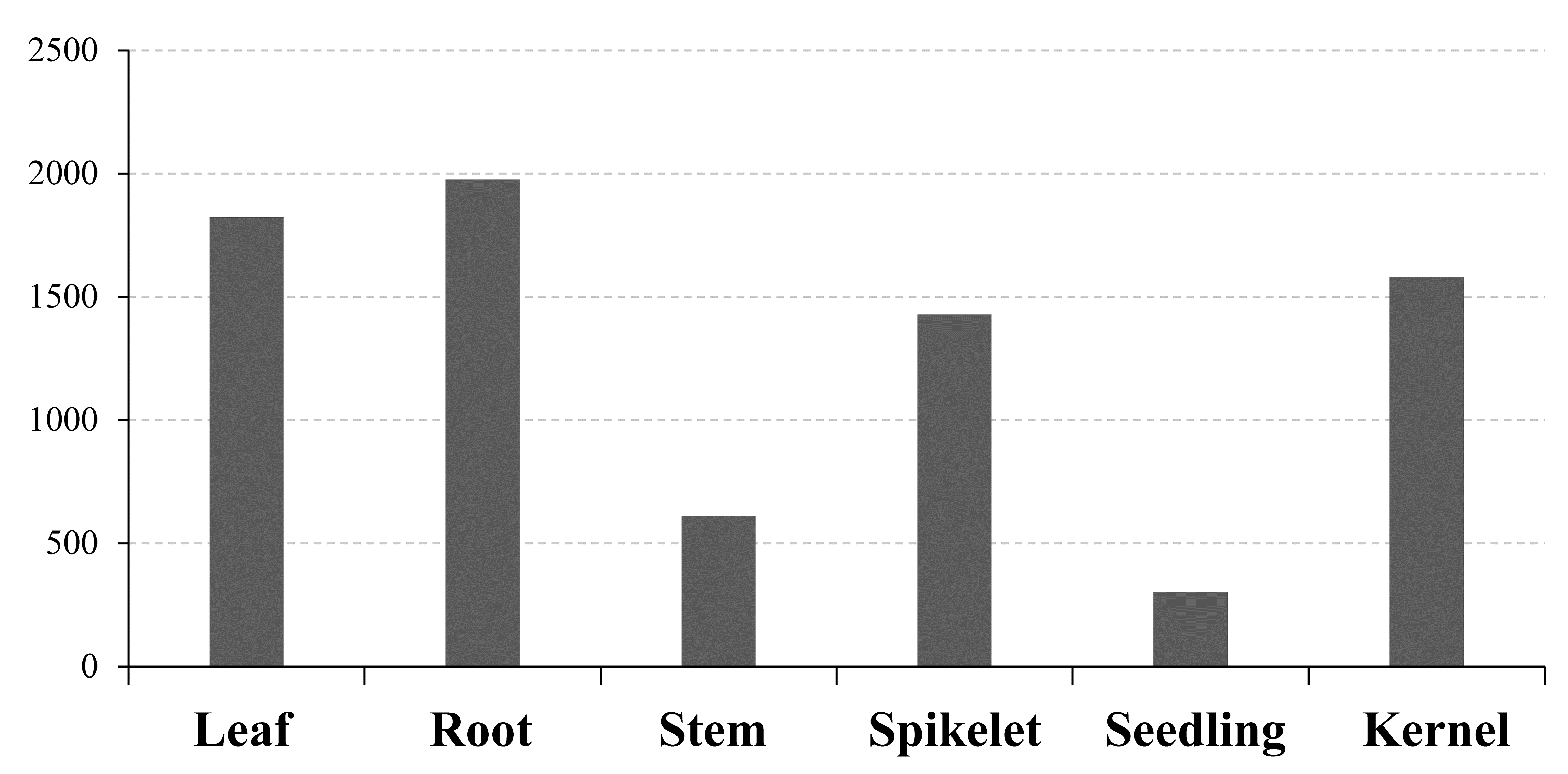


**Fig. S6. Numbers of tissue-specific and highly expressed genes in six tissues.**

**Leaf Root**


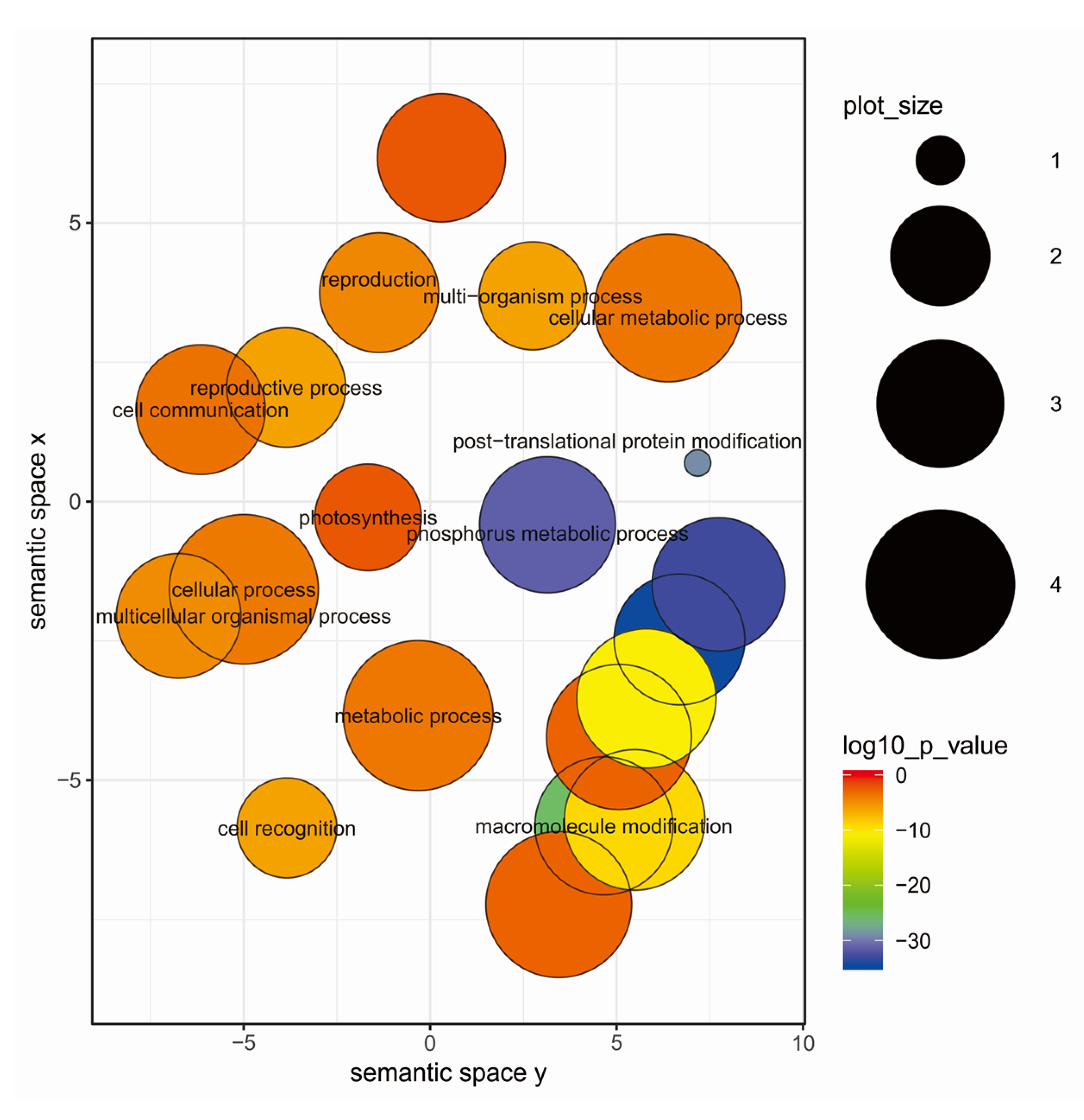

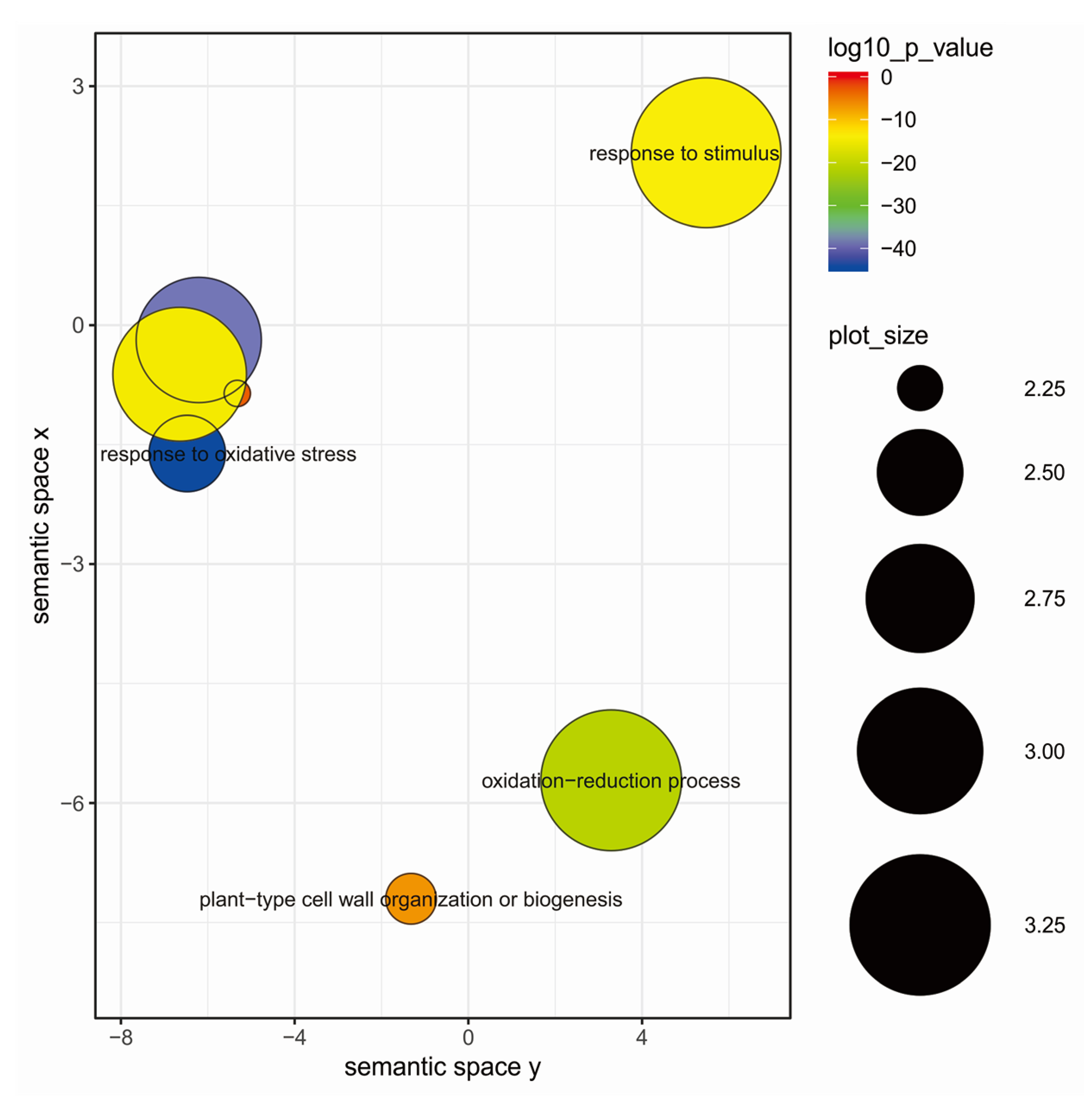


**Kernel Seedling**


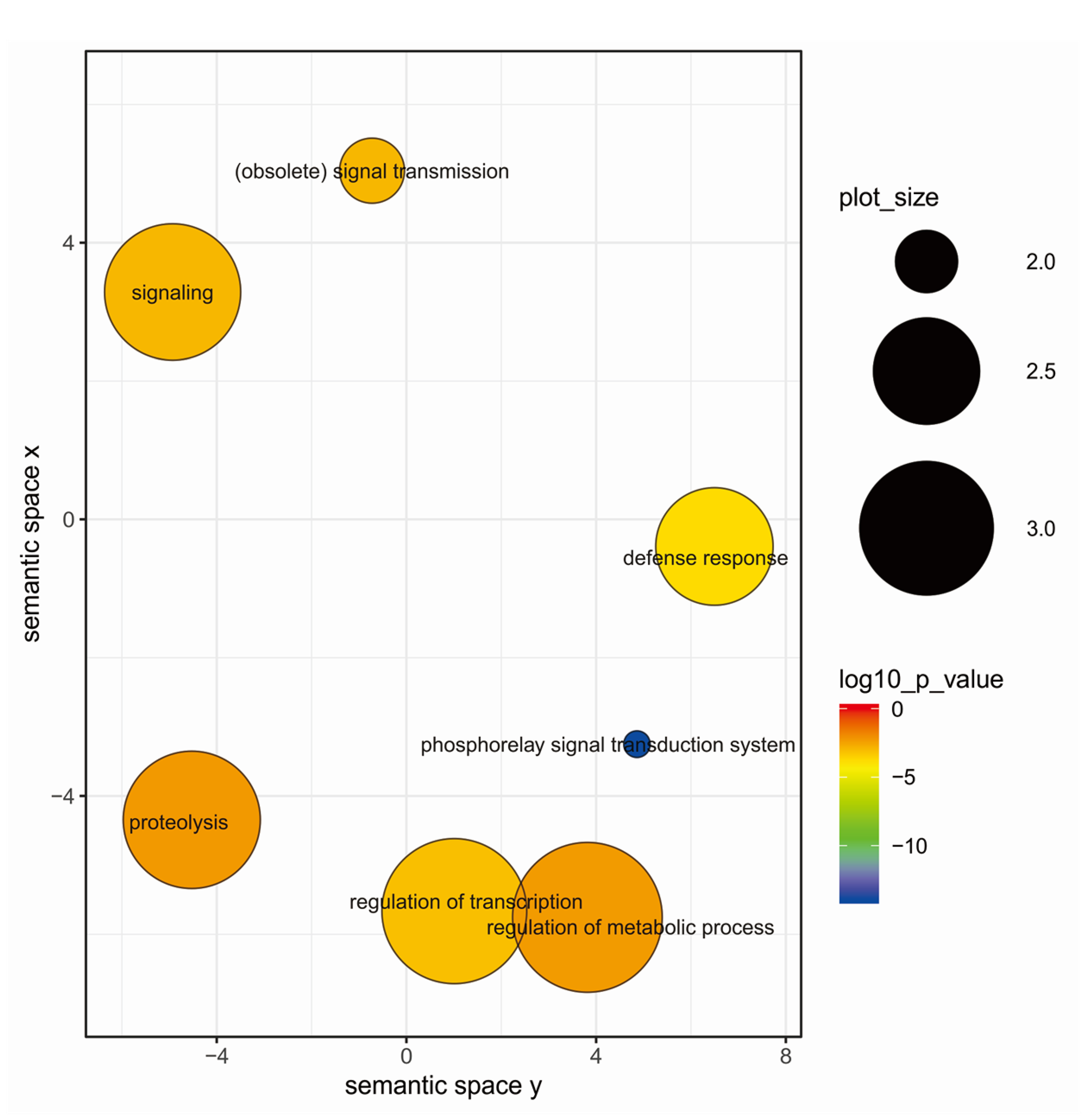

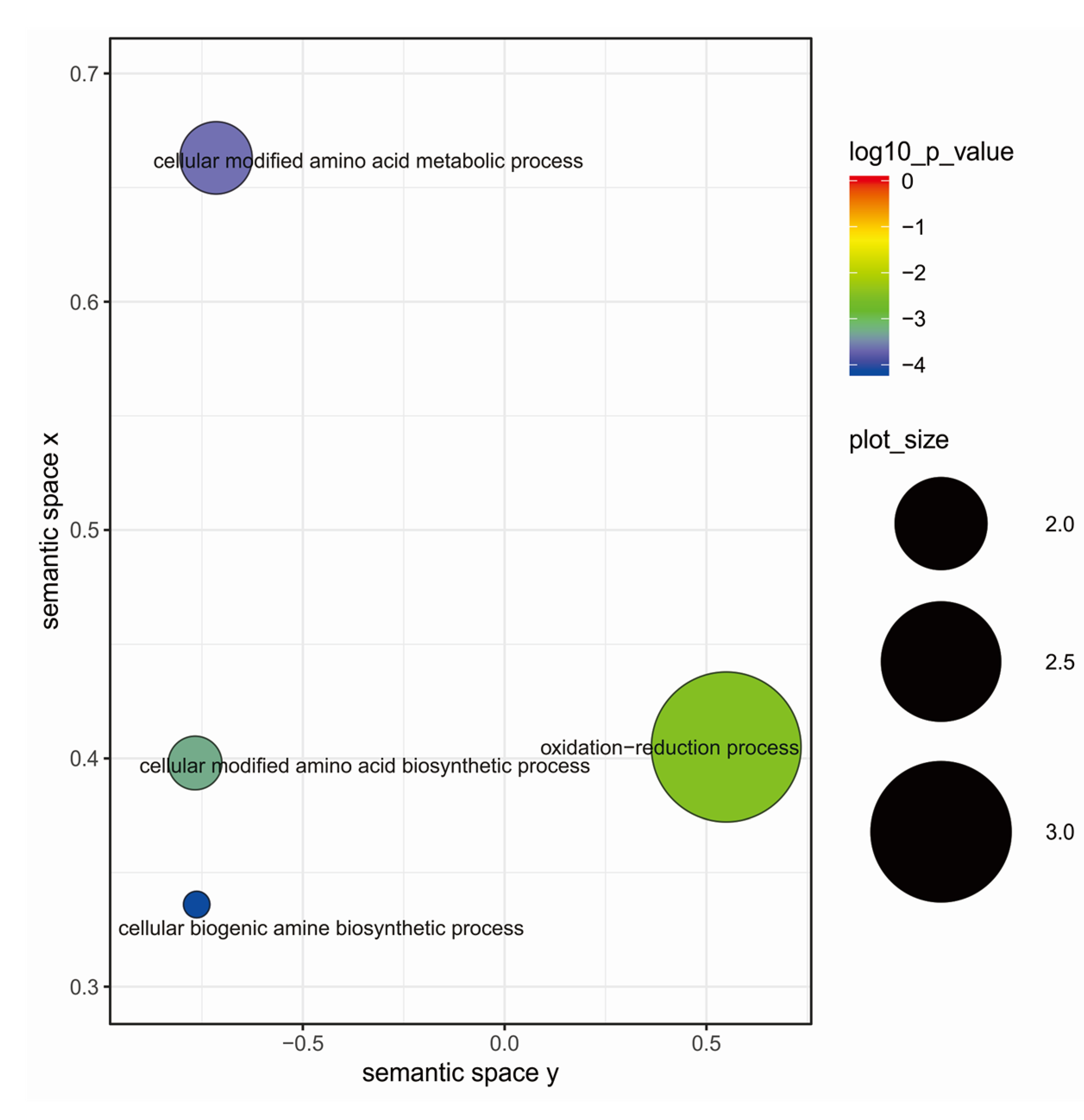


**Spikelet Stem**


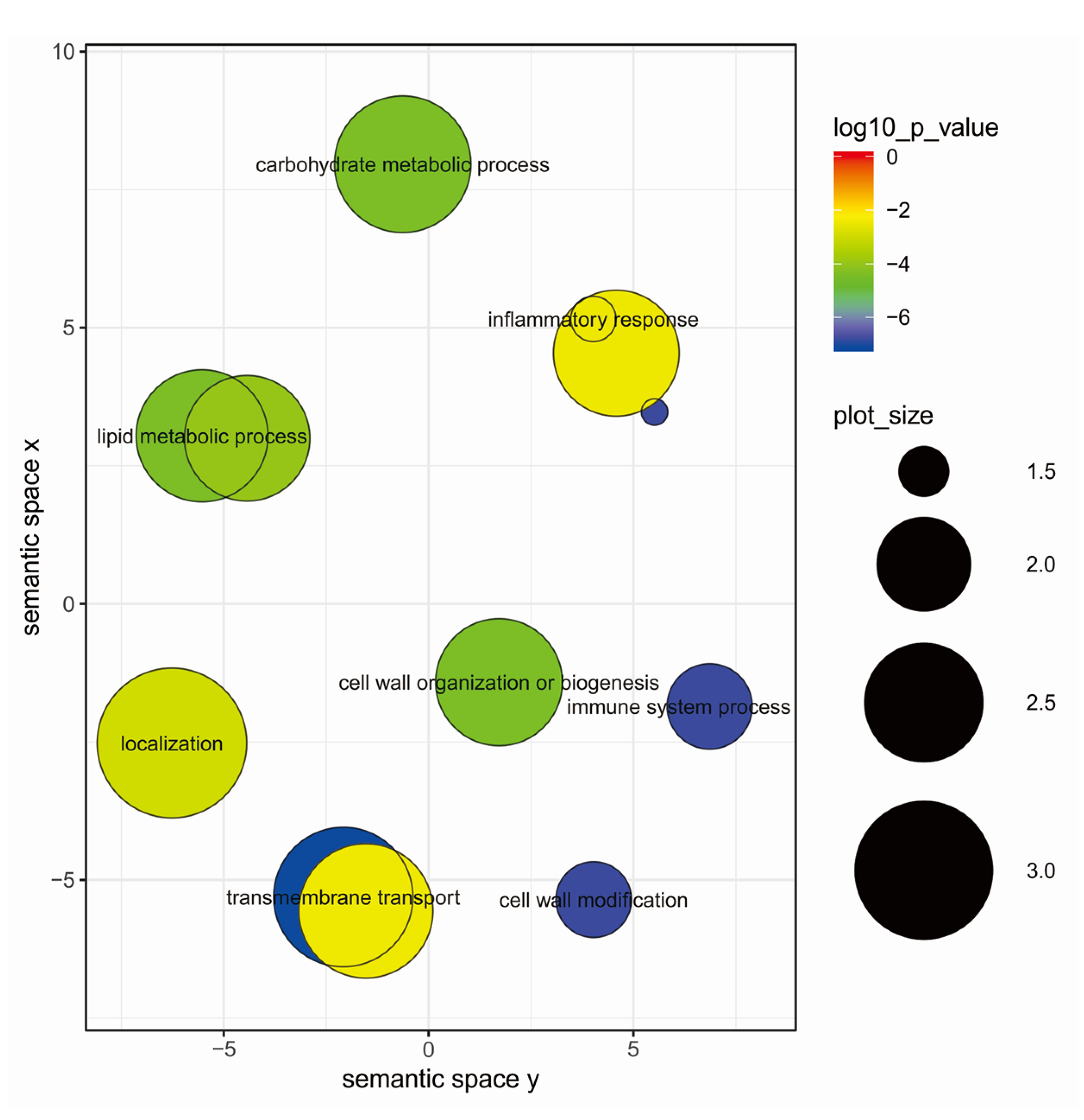

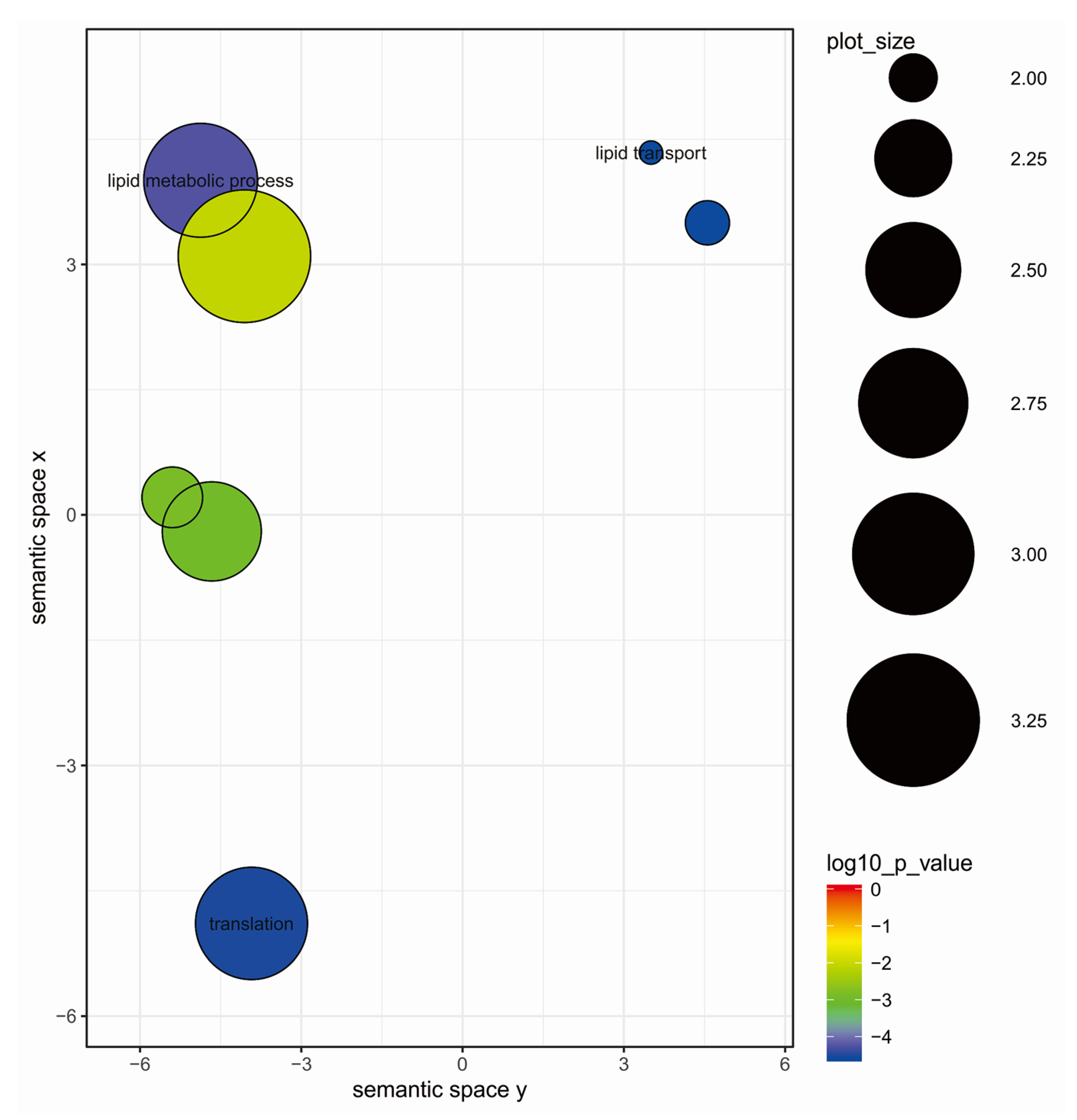


**Fig. S7. GO term enrichment of highly expressed genes in different tissues.** Each circle represents a GO term. Within the Cartesian coordinates (x, y), the closer the circles rest, the more related the GO terms are. The size of the circles is proportional to the number of child GO terms. The circle colour represents the significance of the enrichment.


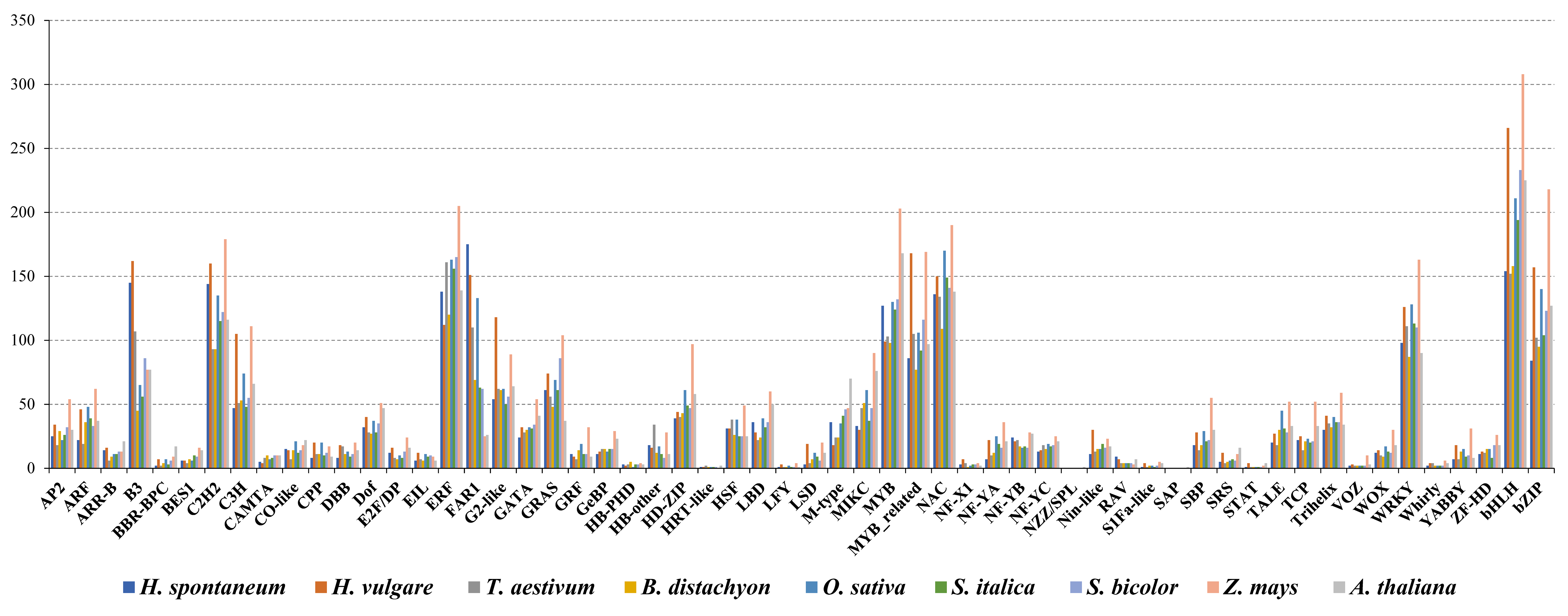


**Fig. S8. Comparison of transcription factor among barley, other grass species and *Arabidopsis*.** Transcription factors of wild barley were predicted with the best hits in *Arabidopsis thaliana* by PlantTFDB (Plant Transcription Factor Database). The data were downloaded from PlantTFDB.

**
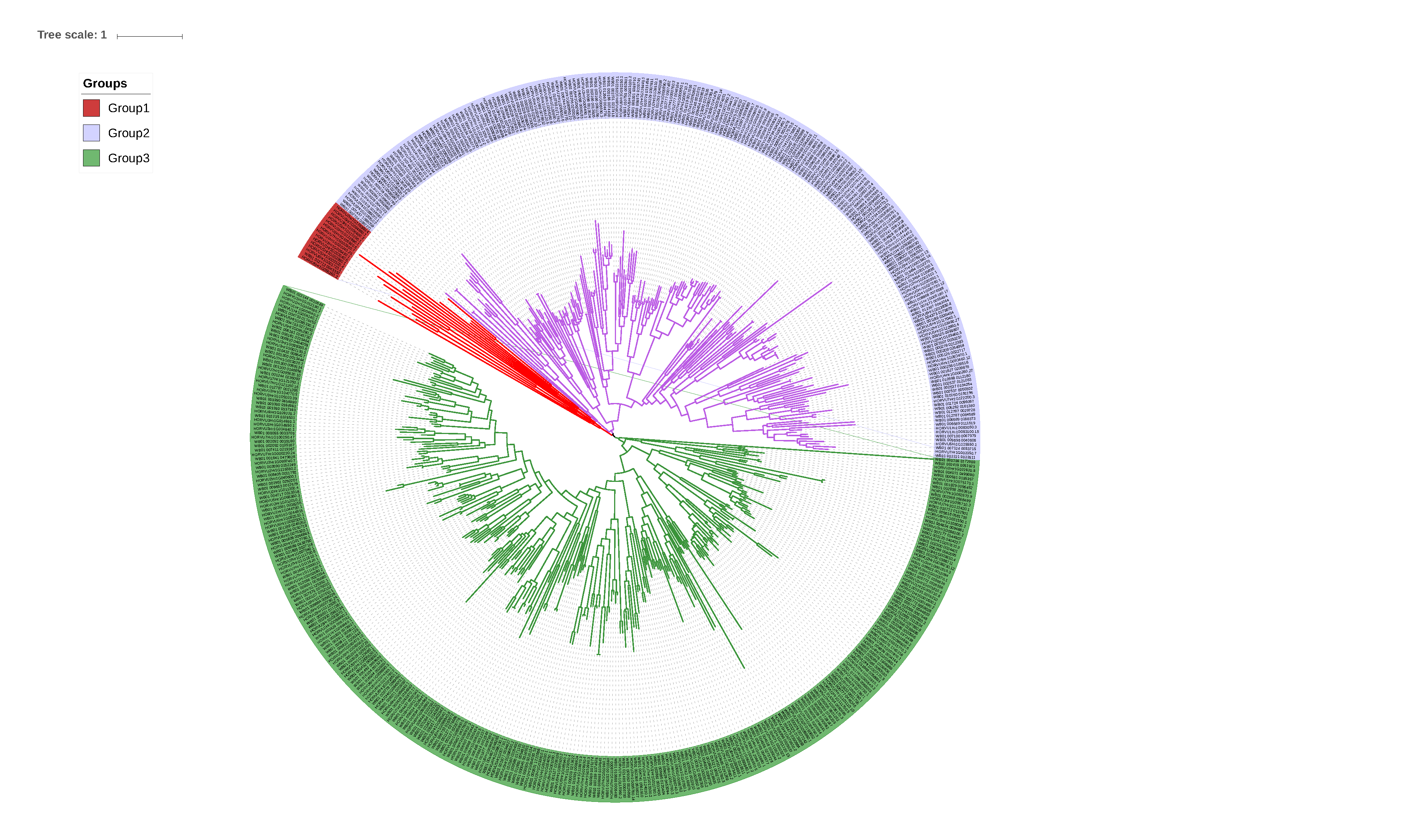
**

**Fig. S9. Phylogenetic analysis of 512 NBS-LRR genes from WB1 and Morex.** The 512 NBS-LRR genes had divided into three main groups: 1) including 15 Morex genes and only 1 WB1 genes (highlighted in red color); 2) including 94 Morex genes and 113 WB1 genes (highlighted in blue color); 3) including 133 Morex genes and 157 WB1 genes (highlighted in green color)
