# Supplementary material for: The draft genome of a wild barley genotype reveals its enrichment in genes related to biotic and abiotic stresses compared to cultivated barley
Source: Plant Biotechnol J. 2019 Aug 4;18(2):443–56. doi: 10.1111/pbi.13210 (PMC6953193; doi:10.1111/pbi.13210)
Supplement: Supplementary file 2 — Table S1 Statistics of the whole genome shotgun sequences of WB1. Table S2 RNA‐seq raw reads of the wild barley genotype WB1. Table S3 De novo assembly of the wild barley genome. Table S4 BUSCO comparison between the wild barley and other published genomes. Table S5 Statistics of repetitive sequences in the wild barley genome. Table S6 A summary of tRNA genes identified in wild barley and its close relatives. Table S7 Families of non‐coding RNA (ncRNA) genes in the wild barley assembly. Table S10 Syntenic loci of the wild barley genotype WB1 on each of the chromosomes of Morex. [file PBI-18-443-s004.doc]

**Additional files:**

**Additional file 2**

**Table S1. Statistics of the whole genome shotgun sequences of WB1.**

| **Library type** | **Insert size** | **No. of paired-end reads** | **Data Yield** | **Sequence depth (X)** |
| --- | --- | --- | --- | --- |
| Paired end | 400 bp | 1,343,073,521 | 402.92 Gb | 93.70 |
| Mate pair | 2.5 Kb | 777,386,776 | 155.47 Gb | 36.16 |
| Mate pair | 9 Kb | 494,237,895 | 98.85 Gb | 22.99 |
| Mate pair | 13 Kb | 240,834,561 | 48.17 Gb | 11.20 |
| **Total** | **-** | **2,855,532,753** | **705.41 Gb** | **164.05** |

**Table S2. RNA-seq raw reads of the wild barley genotype WB1.**

| **Tissues** | **No. of paired-end reads** | **Data Yield** |
| --- | --- | --- |
| Leaf | 69,045,183 | 13.81 Gb |
| Root | 89,925,359 | 17.98 Gb |
| Stem | 90,670,621 | 18.13 Gb |
| Spikelet | 84,955,226 | 16.99 Gb |
| Seed | 85,491,484 | 17.10 Gb |
| Seedling | 90,175,949 | 18.04 Gb |
| Total | 510,263,822 | 102.05 Gb |

**Table S3. *De novo* assembly of the wild barley genome.**

|  | **Contig** | | **Scaffold** | |
| --- | --- | --- | --- | --- |
|  | **size (bp)** | **Number** | **size (bp)** | **Number** |
| **N50** | 35,373 | 31,926 | 724,931 | 1,681 |
| **N60** | 27,639 | 44,738 | 567,152 | 2,349 |
| **N70** | 20,582 | 61,511 | 426,968 | 3,221 |
| **N80** | 13,615 | 85,289 | 299,856 | 4,412 |
| **N90** | 6,408 | 127,224 | 167,395 | 6,294 |
| **Longest** | 473,073 | - | 4,913,581 | - |
| **Total Size** | 4,009,155,661 | - | 4,279,658,192 | - |
| **Total Number (>=200bp)** |  | 478,842 | - | 177,289 |
| **Total Number (>=2kb)** |  | 194,074 | - | 15,508 |

**Table S4. BUSCO comparison between the wild barley and other published genomes.**

| **Species** | **Complete**  **BUSCOs** | **Complete**  **Duplicated**  **BUSCOs** | **Fragmented**  **BUSCOs** | **Missing**  **BUSCOs** | **Reference** |
| --- | --- | --- | --- | --- | --- |
| *H.spontaneum* (WB1) | 95.3% | 16.6% | 2.1% | 2.6% | Current study |
| *H.vulgare* Morex | 93.0% | 15.0% | 2.4% | 4.0% | Mascher et al., 2017 |
| *H.vulgare Haruna Nijo* | 80.2% | 17.1% | 8.6% | 11.2% | Sato et al., 2015 |
| *Ae.tauschii* | 94.5% | 16.0% | 2.8% | 2.7% | Jia et al., 2013 |
| *T.urartu* | 86.3% | 19.0% | 4.3% | 9.4% | Ling et al., 2013 |
| *T.aestivum* | 95.4% | 91.1% | 2.5% | 2.1% | International Wheat Genome Sequencing Consortium, 2014 |
| *B.distachyon* | 95.5% | 14.1% | 2.3% | 2.2% | International Brachypodium Initiative, 2010 |
| *A.thaliana* | 95.7% | 15.6% | 1.4% | 2.9% | Arabidopsis Genome Initiative |
| *O.sativa* | 96.9% | 13.6% | 1.4% | 1.7% | Goff et al., 2002 |
| *S.bicolor* | 95.8% | 14.6% | 1.9% | 2.3% | Paterson et al., 2009 |
| *Z.mays* | 94.8% | 20.4% | 1.7% | 3.5% | Schnable et al., 2009 |
| *S.italica* | 95.9% | 14.2% | 1.9% | 2.2% | Bennetzen et al., 2012 |

**Table S5**. **Statistics of repetitive sequences in the wild barley genome.**

|  | **Length occupied (bp)** | **Percentage of sequences (%)** |
| --- | --- | --- |
| **Class I elements (Retroelements)** | **2,823,198,967** | **65.97** |
| **LTR Retrotransposon** | 2,776,262,656 | 64.87 |
| LTR/Copia | 747,400,753 | 17.46 |
| LTR/Gypsy | 2,025,653,771 | 47.33 |
| unclassified LTR | 3,208,132 | 0.07 |
| **non-LTR Retrotransposon** | 46,936,311 | 1.10 |
| **LINE** | 45,055,936 | 1.05 |
| LINE/L1 | 42,156,369 | 0.99 |
| LINE/R1 | 2,899,567 | 0.07 |
| **SINE** | 1,880,375 | 0.04 |
| SINE/tRNA | 1,176,120 | 0.03 |
| unclassified SINE | 704,255 | 0.02 |
| **Class II elements (DNA Transposons)** | **276,451,346** | **6.46** |
| **DNA Transposon** | 281,065,043 | 6.57 |
| DNA/En-Spm | 241,200,741 | 5.64 |
| DNA/Tourist | 1,570,890 | 0.04 |
| DNA/Harbinger | 8,741,991 | 0.20 |
| DNA/hAT-Tag1 | 806,469 | 0.02 |
| DNA/hAT-Ac | 642,460 | 0.02 |
| DNA/hAT-Tip100 | 207,761 | 0.00 |
| DNA/MuDR | 10,090,665 | 0.24 |
| DNA/TcMar-Stowaway | 10,505,670 | 0.25 |
| unclassified DNA Transposon | 7,298,396 | 0.17 |
| **Unknown repeats** | **232,718,992** | **5.44** |
| **Total transposable elements** | **3,319,190,001** | **77.56** |
| **Small RNA** | **1,514,368** | **0.04** |
| **Satellites** | **11,594,286** | **0.27** |
| **Simple repeats** | **1,045,175** | **0.02** |
| **Total bases masked** | **3,330,988,248** | **77.83** |

**Table S6**. **A summary of tRNA genes identified in wild barley and its close relatives.**

| **Species** | **tRNAs decoding Standard**  **20 AA** | **Selenocysteine tRNAs**  **(TCA)** | **Possible suppressor tRNAs**  **(CTA, TTA)** | **tRNAs with undetermined/unknown isotypes** | **Predicted pseudogenes** | **Total**  **tRNAs** |
| --- | --- | --- | --- | --- | --- | --- |
| *H.spontaneum* AWCS276 | 1340 | 6 | 2 | 35 | 537 | 1920 |
| *H.vulgare*  Morex | 1842 | 0 | 1 | 33 | 417 | 2293 |
| *H.vulgare HarunaNijo* | 1288 | 8 | 3 | 26 | 361 | 1686 |
| *Ae.tauschii* | 2714 | 2 | 0 | 21 | 393 | 3130 |
| *T.urartu* | 2864 | 19 | 4 | 22 | 509 | 3418 |
| *T.aestivum* | 10011 | 8 | 13 | 78 | 1555 | 11665 |
| *B.distachyon* | 593 | 0 | 0 | 7 | 15 | 615 |
| *A.thaliana* | 685 | 0 | 0 | 1 | 13 | 699 |
| *O.sativa* | 722 | 0 | 0 | 0 | 26 | 748 |
| *S.bicolor* | 577 | 1 | 1 | 8 | 62 | 649 |
| *Z.mays* | 1440 | 4 | 7 | 13 | 832 | 2296 |
| *S.italica* | 634 | 0 | 0 | 18 | 58 | 710 |

**Table S7. Families of non-coding RNA (ncRNA) genes in the wild barley assembly.**

| RNA type | Number in wild barley | Average length(bp) | Total length(bp) |
| --- | --- | --- | --- |
| tRNA | 1,920 | 73 | 140,489 |
| miRNA | 558 | 141 | 78,827 |
| snRNA | 136 | 156 | 21,263 |
| CD-box RNA | 77 | 140 | 10,755 |
| HACA-box RNA | 86 | 306 | 26,351 |

**Table S10**. **Syntenic loci of the wild barley genotype WB1 on each of the chromosomes of Morex.**

| **Morex** | **No. of**  **syntenic gene**  **blocks** | **No. of**  **syntenic**  **genes** | **Average gene**  **number per**  **block** |
| --- | --- | --- | --- |
| Chromosome-01 | 208 | 1,239 | 6 |
| Chromosome-02 | 257 | 1,601 | 6 |
| Chromosome-03 | 254 | 1,669 | 7 |
| Chromosome-04 | 213 | 1,295 | 6 |
| Chromosome-05 | 203 | 1,338 | 7 |
| Chromosome-06 | 198 | 1,059 | 5 |
| Chromosome-07 | 225 | 1,517 | 7 |
| **Sum** | **1,558** | **9,718** | **6** |

**Reference:**

Arabidopsis Genome Initiative. Analysis of the genome sequence of the flowering plant Arabidopsis thaliana. Nature. 2000;408(6814):796.

Bennetzen JL, Schmutz J, Wang H, Percifield R, Hawkins J, Pontaroli AC, et al. Reference genome sequence of the model plant Setaria. Nature Biotechnol. 2012;30(6):555.

Goff SA, Ricke D, Lan TH, Presting G, Wang R, Dunn M, et al. (). A draft sequence of the rice genome (Oryza sativa L. ssp. japonica). Science. 2002;296(5565):92-100.

International Brachypodium Initiative. Genome sequencing and analysis of the model grass Brachypodium distachyon. Nature. 2010;463(7282):763.

International Wheat Genome Sequencing Consortium. A chromosome-based draft sequence of the hexaploid bread wheat (Triticum aestivum) genome. Science. 2014;345(6194):1251788.

Jia J, Zhao S, Kong X, Li Y, Zhao G, He W, et al. Aegilops tauschii draft genome sequence reveals a gene repertoire for wheat adaptation. Nature. 2013;496(7443):91.

Ling HQ, Zhao S, Liu D, Wang J, Sun H, Zhang C, et al. Draft genome of the wheat A-genome progenitor Triticum urartu. Nature. 2013;496(7443):87.

Mascher M, Gundlach H, Himmelbach A, Beier S, Twardziok SO, Wicker T, et al. A chromosome conformation capture ordered sequence of the barley genome. Nature. 2017;544(7651):427-433.

Paterson AH, Bowers JE, Bruggmann R, Dubchak I, Grimwood J, Gundlach H, et al. The Sorghum bicolor genome and the diversification of grasses. Nature. 2009;457(7229):551.

Sato K, Tanaka T, Shigenobu S, Motoi Y, Wu J, Itoh T. Improvement of barley genome annotations by deciphering the Haruna Nijo genome. DNA Res. 2015;23(1):21-28.

Schnable PS, Ware D, Fulton RS, Stein JC, Wei F, Pasternak S, et al. The B73 maize genome: complexity, diversity, and dynamics. Science. 2009;326(5956):1112-1115.
